# Acknowledgment to Reviewers of *JCM* in 2021

**DOI:** 10.3390/jcm11030807

**Published:** 2022-02-03

**Authors:** JCM Editorial Office

**Affiliations:** MDPI AG, St. Alban-Anlage 66, 4052 Basel, Switzerland

Rigorous peer-reviews are the basis of high-quality academic publishing. Thanks to the great efforts of our reviewers, *JCM* was able to maintain its standards for the high quality of its published papers. Thanks to the contribution of our reviewers, in 2021, the median time to first decision was 20 days and the median time to publication was 40 days. The editors would like to extend their gratitude and recognition to the following reviewers for their precious time and dedication, regardless of whether the papers they reviewed were finally published:
A. Robles-MarhuendaKazutaka KamiyaA. SchlittKazuto TajiriA. V. RakovKazuya InokiAalap ChokshiKazuyuki HirookaAarno DietzKe WangAaron E. EmbryKei EndoAatke Van Der MaasKei MoriyaAbayomi T. OgunjimiKei SaitoAbbey SchepersKeigo UchimuraAbdelaali HassaineKeiichi HironoAbdelahhad BarbourKeiichi KubotaAbdo E. KabarritiKeiichi MatsubaraAbdul JabbarKeiichi TominagaAbdullah Al ShoyaibKeiichiro UeharaAbeer Al-GharaibehKeiko IshigakiAbheepsa MishraKeiko YoshimotoAbhilash PerisettiKeishi FujioAbhilash VenugopalanKeiso TakahashiAbhirup DasKeissy SousaAbhishek MaitiKeisuke IzumiAbigail I. WaldKeita KouzuAbigail L. WhitehouseKeith HansenAbinaya Priya VenkataramanKeith WongAbirami KirubarajanKelechi NjokuAbish StephenKelli W. WilliamsAboozar MonavarfeshaniKelly ByrneAbraham DibKelly DomvriAchille MarinoKelly SchieltzAchilleas BoutsiadisKelly Virecoulon GiudiciAchim BurrerKelsey Needham DancauseAcke OhlinKelton P. TremellenAda W.S. LeungKen KuduraAdam BlumenbergKen Kuljit S. ParharAdam KernKen TakasawaAdam KlocperkKen WashioAdam LewisKengo FujiiAdam M DubisKenichi YamadaAdam ObrKenichiro HiraokaAdam SheppardKenichiro MotomuraAdam TrepczynskiKenji HirataAdam WhitleyKenji KawaiAdam WichniakKenji TsuboshimaAdam WylęgałaKenneth A. AndreoniAdamanthia LiapikouKenneth BlumAdele MitrottiKenneth Dürsteler MacFar-landAdi LadorKenneth HungAdi LahatKenneth Wayne AltmanAdi Laurentiu TarcaKennio Ferreira-PaimAdi MehtaKensuke SasakiAditi BhargavaKentaro IwataAditi SinghviKentaro OhkoAditi SwarupKentaro WatanabeAditya GoudKenya KusunoseAdmed ElamirKerstin RohdeAdnan KhanKesava ReddyAdolf LukanovićKeshav GopalAdolfo Baloira VillarKeshav KohliAdolfo Di FioreKeti VitanovaAdriaan Anthonie Van Bode-gravenKetil HeimdalAdrian ArrietaKeunBaDa SonAdrian CurtoKeun-Yeong JeongAdrian DalyKevin BakerAdrian GajewskiKevin CoulouresAdrian GombartKevin HoAdrian Kah Heng ChiowKevin J HsuAdrian Noriega De La ColinaKevin J. LandAdrian OoKevin L. GarvinAdrian T. BilleterKevin LachapelleAdrian WaggKevin LobdellAdrian WlodarczakKevin N. GriffithAdriana C. PanayiKevin RoedlAdriana DulameaKevin RolfeAdriana RomitiKevin StaatsAdrien FlahaultKeyla Vargas-RománAdwitia DeyKfir SharabiAffan ShoukatKhaled QanudAfona ChernetKhalid ShoumariyehAfsane RiaziKhalil ZamanAftab AlamKhodabakhsh JavanshirAgata GabryelskaKhodr TelloAgata JanowskaKhristine Joy C. AntiguaAgata SzulcKi Ho ParkAgata Wawrzkie-wicz-JałowieckaKi-cheong ParkAglaja StirnKieran O'SullivanAgnė Laučytė-CibulskienėKihye HanAgnès LefortKikuo IsodaAgnès MeybeckKilian Martin GustAgnese PoKilian WeigandAgnieszka A. Bojarska-JunakKim BlakeAgnieszka BartoszekKimber SimmonsAgnieszka ChwilkowskaKimberly L. RayAgnieszka Cud-noch-JȩdrzejewskaKimberly PerezAgnieszka Dettlaff-PokoraKiminobu TanizawaAgnieszka Gala-BłądzińskaKimitoshi YagamiAgnieszka GniadekKinda IbrahimAgnieszka KotalczykKinga Grzech-LesniakAgnieszka Lecka-AmbroziakKinga Kowalska-DuplagaAgnieszka Mün-ster-WandowskiKinga MusiałAgnieszka NawrockaKira BacalAgnieszka Owczar-czyk-SaczonekKiran KarunakaranAgnieszka PawlakKiran SandhuAgnieszka SkoczylasKirill LyapichevAgnieszka Wiśniowska-SzurlejKirsi M. IsoherranenAgostino GaudioKirsten AhringAgueda González RodríguezKirsten H. LimesandAgustín Fernández-CisnalKirstin VachAhmad Al-MrabehKirtikar ShukiaAhmad M. SlimKirtikar ShuklaAhmad MirzaKishan Kumar NyatiAhmed A. HusseinKishor GanganiAhmed AbdallaKishu RanjanAhmed Abdel Khalek Abdel RazekKissy Guevara-HoyerAhmed AbdelghanyKisuk MinAhmed AbuzaidKivovics MártonAhmed ElamragyKiwamu MatsuokaAhmed H. MekkawyKi-won MoonAhmed IsmaeelKiyohide IshihataAhmed SawasKiyoshi MaedaAhmet BindayiKiyoshi MoriAida PetcaKiyoshi UemasuAiham QdaisatKiyotaka UchiyamaAimee Di MarcoKiyotake IshikawaAimen KhacharemKjell Å SalvesenAimilia Lydia KalafateliKlara RostaAino SiltariKlaudiusz NadolnyAisha MiahKlaus EmmanuelAisling E CourtneyKlaus KroghAjay AshokKlaus W. EichhornAjay Kumar MishraKlearchos PsychogiosA.J.M. LigtenbergKlemens BuddeAkhil VarshneyKliment GatzinskyAki KoivulaKnut Asbjørn Alexander ØymarAkihiko HiyamaKnut LundinAkihiro KuwahataKo OkumuraAkihiro SunagaKoen De SchrijverAkihiro YasumotoKoichi OtaniAkiko EguchiKoichiro FujisueAkiko TodakaKoichiro KawaguchiAkinari SawadaKoji AndoAkinobu SuzukiKoji NishimuraAkio HoriguchiKoji OtaniAkira DobashiKoji TakahasiAkira HondaKoji YokoyamaAkira KodamaKok Yong ChinAkira MatsushimaKolja FreierAkira MonjiKondru NaveenAkira YamamiyaKonosuke NakajiAkitatsu HayashiKonstantin DobererAkiyoshi MatsugiKonstantin KrasagakisAkram MohammedKonstantina StathopoulouAkram ZaaqoqKonstantinia AlmpaniAla NozariKonstantinos A. GatzoulisAlain CalenderKonstantinos AvgerinosAlain FreyKonstantinos DimopoulosAlan BonderKonstantinos DitsiosAlan EmondKonstantinos G. KyriakoulisAlan G. FinkelKonstantinos KatogiannisAlan KaplanKonstantinos LasithiotakisAlan Neil SchechterKonstantinos PontikisAlan T BlankKonstantinos SapalidisAlarico ArianiKonstantinos StergiosAlastair N. GossKonstantinos TsioufisAlauddin BhuiyanKonstanty SzułdrzyńskiAlba Martín-GilKoo Jeong KangAlba Sánchez-TorresKornelia JaquetAlbert F. MoraskaKosei NagataAlbert FiguerasKosho YamanouchiAlbert J. FenoyKosmas DaskalakisAlbert RogersKosuke MinagaAlbert WabneggerKotaro TsuboiAlberta A. H. J. ThiadensKozo OkadaAlberto Adanero VelascoK.R RamanathanAlberto AimoKrediet Raymond TheodorusAlberto BerenguerKripa ShankarAlberto CiprianiKrishan K. PandeyAlberto ClementeKrishna Kishore UmapathiAlberto Diniz-FilhoKrishna Mohan SurapaneniAlberto DolciKrishna Prahlad MaremandaAlberto FerrareseKristen A McLaurinAlberto Garcia-OrtegaKristen M. WardAlberto J. SchuhmacherKristen RakAlberto LapiniKristijan SkokAlberto López-LeraKristin E. MusselmanAlberto MellaKristin HauganAlberto PalazzuoliKristina Pogrmic‐MajkicAlberto Quílez-RobresKristine RuppertAlberto RebonatoKristoffer StrålinAlberto RomanoKristy WittmeierAlberto SignoreKrupal B. PatelAlberto VerrottiKruthika ThangaveluAlberto ZamoraKrystel Nyangoh-TimohAlbino EccherKrystyna OlczykAlbree Tower-RaderKrzysztof DomagalskiAldo ClericoKrzysztof DyrbuśAldo RoccaKrzysztof GałczyńskiAldo ScafoglieriKrzysztof GorynskiAldona SiennickaKrzysztof J. CzarnockiAlejandra Cardenas-RojasKrzysztof KobielakAlejandra ConsejoKrzysztof KrystaAlejandro De FeriaKrzysztof MorawskiAlejandro Ibáñez-CostaKrzysztof MuchaAlejandro Santos-LozanoKrzysztof OrczykAlejandro Solís PeñaKrzysztof OzierańskiAlejandro Suarez-De-La-RicaKrzysztof SmarzAleksander AraszkiewiczKrzysztof SzklannyAleksander ChaibiKrzysztof SztankeAleksander KuśKsenija Rener-SitarAleksandra FucicKubra AralAleksandra Gi-lis-JanuszewskaKuldeep Singh AttriAleksandra Kar-czmarska-WódzkaKuljit SinghAleksandra KołotaKumar JayantAleksandra Majchrzak-CelińskaKunal DhimanAleksandra Nitecka-BuchtaKunal VakhariaAleksandra Piecho-ta-PolanczykKun-Bo ParkAleksandra SędzikowskaKunihiko HiramatsuAleksandra SzczepankiewiczKunito OkuyamaAleksandra SzylińskaKunwei WuAleksandra VuckovicKuo-Yao HsuAleksejus ZorinasKurosch YazdiAleksey Michailovich ChaulinKwang LimAlem MehariKwanghyun LeeAlena OrlenkoKwang‐No LeeAlenka ŠkerjancKye Hun KimAleš TomažičKyeong-tae LeeAlessandra AldieriKyle BurghardtAlessandra LaricchiaKyle PoulsenAlessandra LintasKyohei MiyamotoAlessandra M. FerraroKyong IhnAlessandra MagentaKyonghwa KangAlessandra MagnaniKyriakos OikonomouAlessandra MarchesiKyu Sup LeeAlessandra PutrinoKyulee ShinAlessandro A. JammalKyung Lhi KangAlessandro AgostiniKyungjin KimAlessandro AlaimoL. M. Gonzalez PerezAlessandro AnselmoL. SchifferAlessandro AntonelliLadislav BatalikAlessandro ArenaLadislav ŠtěpánekAlessandro ArrigoLaia Fernández-BaratAlessandro BellettiLaith R SultanAlessandro CastiglioniLampros K. MichalisAlessandro CicolinLampros MitrakasAlessandro ComandoneLana Antoinette CastellucciAlessandro FascianiLanfranco PellesiAlessandro FavilliLara GianeselloAlessandro GalaniLara McClureAlessandro GambellaLara Valiño-RivasAlessandro GonfiottiLaraine WinterAlessandro GranitoLarisa MorosanAlessandro LeonidaLarissa A. McGarrityAlessandro MalobertiLars Bo SvendsenAlessandro MantovaniLars EckardtAlessandro MathieuLars GrøvleAlessandro MeduriLars HeggelundAlessandro PirasLars Henrik FrichAlessandro PreteLars Vedel KessingAlessandro PusateriLarsen Erik RojAlessandro RizzoLashitew GedamuAlessandro ScalaLászló SzilákAlessandro VittoriLauge ØstergaardAlessandro ZampognaLaukhtina EkaterinaAlessandro ZerbiLaura AcquasalienteAlessia ArcaroLaura Blanco-HinojoAlessia FinottiLaura BurattiAlessia PedotoLaura CannavòAlessia RubiniLaura CortiAlessio ArdizzoneLaura DellazizzoAlessio ArriviLaura ForbesAlessio GasperettiLaura Francesca PisaniAlessio MazzoniLaura FranzaAlessio MetereLaura GianottiAlessio MylonasLaura GilbertsonAlessio PaladiniLaura Giuseppina Di PasquaAlessio RungatscherLaura HemmyAleth PerdrigerLaura KaravirtaAlex AdesLaura ManciniAlex Al KhouryLaura MangiaviniAlex Almuedo RieraLaura MilazzoAlex B. BlairLaura MolesAlex BertramsLaura Morales-FernandezAlex C. VargheseLaura Mouriño-AlvarezAlex CuencaLaura Muino MosqueraÁlex FerrerLaura PeraltaAlex MirnezamiLaura RigonAlex SoldererLaura SanapoAlex SparreboomLaura Torres-ColladoAlexa Karina KlettnerLaura TurcoAlexander Achalanda-baso-OchoaLaura VicenteAlexander B. A. VonkLaure Sarda-MantelAlexander BerezinLauren K. WarehamAlexander BurashnikovLauren R. AlesiAlexander DomnichLaurence DedekenAlexander G. TruesdellLaurent AudigéAlexander GiureaLaurent BlaironAlexander H. MaassLaurent De LandsheereAlexander HaraganLaurent MetzingerAlexander K. SchusterLazzaro ParaggioAlexander KaltenboeckLech PaluszkiewiczAlexander KleinLee L. SwanströmAlexander MusteaLee Samüel WannAlexander PodgorsakLee Yee Han DaveAlexander PottLeesa Van NiekerkAlexander ScheiterLei HuoAlexander SedaghatLeif C. AnderssonAlexander TamalunasLeila RahnamaAlexander V. ProninLela BuckinghamAlexander WaldthalerLena HirtlerAlexander YounsiLena Nilsson-WikmarAlexander ZimmererLenar Tatios YessayanAlexander ZoufalyLenard ConradiAlexander-N. ZellerLene Ring MadsenAlexandra ArvanitakiLenze Nicholas R.Alexandra BöhmLeo L. TsaiAlexandra DipperLeon DinshawAlexandra GratlLeon Van KempenAlexandra I. StavrakisLeonard BieloryAlexandra LjimaniLeonardo FabbriAlexandra M. SminkLeonardo Jose Mata-runa-Dos-SantosAlexandra MateiLeonardo ManciniAlexandra PerricosLeonidas PalaiodimosAlexandra SmithLeonor ArenillasAlexandra Vink-NieseLeonor Bacelar-NicolauAlexandre BalaphasLesley Ann SaketkooAlexandre BodinLeslie Robinson‐BostomAlexandre Docampo‐SimónLeszek BryniarskiAlexandre E. MalekLeszek KrólickiAlexandre EscandeLetizia DeantonioAlexandre GaymardLev ShvidelAlexandre JoostenLewis HsuAlexandre RouxLewis Mehl-MadronaAlexandros G. SykarasLi LiAlexandros LaiosLi Lieber Po-HungAlexandros PatrianakosLi YumanAlexandros RovasLiam WignallAlexandru Bogdan CiubaraLiana PlesAlexandru BurlacuLiang-Chun ShihAlexandru UliciLiang-Tseng KuoAlexey MeshkovLiangwan ChenAlexey SarapultsevLibor VelisekAlexis Ceecee ZhangLicun WuAlexis RuetLidija GradišnikAlexis UlrichLiisa LehtonenAlfonso AntónLijckle Van Der LaanAlfonso GalderisiLiliana Szyszka-SommerfeldAlfonso LorenzoLilong GuoAlfredo Abad-GurumetaLimongi FedericaAlfredo CampennìLimor Muallem-KalmovichAlfredo CaturanoLina A. ShehadehAlfredo IandoloLina BergmanAlfredo R. GalassiLina Holm IngelsrudAlfredo TartaroneLina SchifferAli Al KaissiLina Ya'QoubAli H. AldoukhiLina YoussefAli Mahdavi FardLinbo ZhaoAli MobasheriLinda HammerichAli Reza KamaliLinda HersheyAli WeinsteinLinda WammesAlice BaronciniLindquist IngridAlice BoilèveLindsay R. FreudAlice BonomiLindsay TetreaultAlice VerticchioLindsey EdwardsAlicia De PabloLindsey KennedyAlicia HeathLine KibsgaardAlícia Molina-AndújarLingli ZhouAlicja Ewa RatajczakLingpeng ShanAlicja NowickaLinh Thuy NguyenAliki-Eleni FarmakiLionel RebiboAlina DimaLior FuchsAlīna SultanovaLirios DuenãsAlina VedutaLisa AdamsAlina WilkowskaLisa CunninghamAline Rangel-PozzoLisa DellAlisa D. KjaergaardLisa E.E.L.O. LashleyAlisa J. Stephens-ShieldsLisa ImundoAlisha HoltzhausenLisa LetzkusAlison SchwartzLisa LoughneyAlistair V. W. NunnLisa LungaroAlix YoungLisa NewsonAljaz HojskiLisa StoryAlkiviadis VatopoulosLisa Tedesco TriccasAllan GottschalkLisa ZondlerAllan PaceyLisandro Miguel MontorfanoAllan R MartinLise BoussemartAllegra BattistoniLisethe MeijerAllison B. ReissLiudmila I. Gerasi-mova-MeigalAllison J. BurbankLivia LamartinaAllister GibbonsLivia ManzellaAlok KumarLivia VeressAlon FarfelLiviu SteierAlp UcokLiza JohannessonÁlvaro Astasio PicadoLjubica OdakAlvaro Rojas-PeñaLluís BassasAlvin J. X. LeeLois A. DaamenAlzira AlmeidaLola Jade PalmieriAmaju A. IkomiLola MugishoAmanda Lopez-PicadoLola NeufcourtAmanda M. EckerLong ShenAmanda RobertsLoredana MoroAmankeldi SalybekovLorena Vega-ZelayaAmany SholkamyLorenzo BacigalupoAmbooj TiwariLorenzo BertaniAmbra GentileLorenzo BrognaraAmedeo LonardoLorenzo CarnevaleAmelia CarettoLorenzo CostaAmelia J. HessheimerLorenzo FalsettiAmika MoroLorenzo NeviAmir EmamifarLorenzo PelusoAmir ShemiraniLorenzo RinaldoAmirbabak Sioofy-KhojineLorenzo Roberto SuardiAmit ChandelLorenzo ScappaticcioAmit PursnaniLorenzo VassalloAmit SegevLorraine A.T. BoakyeAmna ZafarLorys CastelliAmosy M'KomaLothar LilgeAmritlal MandalLouis ArnouldAmy DeZernLouis KreitmannAmy E. HarwoodLouis P. PerraultAmy L ShaverLouis Philippe LaurinAmy R BlandLouis PuybassetAmy Tu WangLouisa MurdinAmy TybergLouise GrahnemoAna AdanLouise McCabeAna AiresLouise SalomoAna Amorim-de-SousaLourdes Luceño-MorenoAna BagüésLu HuangAna BanitoLuc FavardAna BlascoLuc Int PanisAna Corte-RealLuc MorinAna DascaluLuc RubingerAna E. Rodríguez-VicenteLuc Van BortelAna Esteve-SoleLuca AmbrosioAna Fernández-AraqueLuca ArcariAna Ferrández MonteroLuca BedettiAna FrancoLuca BertolacciniAna GorostidiLuca BrazziAna Guadaño-FerrazLuca BruschiniAna Isabel Alvarez-MercadoLuca CamoniAna Isabel Corregi-dor-SanchezLuca CevolaniAna Isabel Sánchez-CanoLuca De SimoneAna Julia García-MalinisLuca Di GianfrancescoAna KezicLuca DiamantiAna KonvalinkaLuca EspositoAna Maria BugăLuca FranchinAna María SerradorLuca GiannellaAna Patricia AndradeLuca Giovanni LocatelloAna R. CostaLuca OrtensiAna Rita FariasLuca QuartuccioAna RochaLuca RevelliAna RufinoLuca RicciardiAna Teresa Almeida-SantosLuca Rosario LimiteAna VukovicLuca SabaAnaïs HausvaterLuca Salvatore De SantoAnan YounisLuca SpieziaAnand SharmaLuca TabbìAnand SinghLuca ValerioAnastasia HyrinaLuca ZanoliAnastasia KirillovaLucas H. P. BerntsAnastasia PavlidouLucatelli PierleoneAnastasia PonasenkoLucca LachetaAnastasia SaadeLucia Carmela CosenzaAnastasia UssiaLucia MangoneAnastasiia I. PetushkovaLucia MerolleAnastasios E. GermenisLucia MirabellaAnastasios KolliasLucia ScisciolaAnastasios KoulaouzidisLucia TricaricoAnastasios KyriazoglouLuciana CacciottolaAnastasios ManiakasLuciana LabancaAnastasiya A. SokolovaLuciano De BiaseAnda BulargaLucilla CrudeleAnder VergaraLucio RovatiAnder Vergara AranaŁucja BieleninikAnders BoydLucrecia Carrera QuintanarAnders C. BoydLucrezia BertinoAnders GottsäterLucrezia TognoloAnders HenricsonLucy A. CouplandAnders LindahlLucy D. MastrandreaAnders S. HenricsonLucy NorrisAndi KrumbholzLucyna OstrowskaAndoni Carrasco-UribarrenLudivine DionAndra MesterLudovica NucciAndrás BallaLudovico MuziiAndre IgneeLuigi A. PastoreAndrea AbateLuigi Alberto PiniAndrea AlexandreLuigi BennardoAndrea Ascoli MarchettiLuigi BrunettiAndrea BarbieriLuigi CarboneAndrea BarisonLuigi CerboneAndrea ButeraLuigi CirilloAndrea CafarelliLuigi LoscoAndrea CarsettiLuigi MeccarielloAndrea CarugnoLuigi SantacroceAndrea CassoniLuigi TaraniAndrea ColliLuigi TritapepeAndrea ConaLuigi TurcoAndrea CoppolaLuigi VetrugnoAndrea CortegianiLuis Andreu CaravacaAndrea CozziLuis AyerbeAndrea D. OlmsteadLuís Carlos MatosAndrea De GiacomoLuis Ceballos-LaitaANDREA De VitoLuis Corral-GudinoAndrea Del CampoLuis F. Gonzalez-CiccarelliAndrea Del GiudiceLuis Gómez PérezAndrea DeregibusLuis Ignacio Gonzá-lez-GranadoAndrea FabbriLuis M BeltránAndrea FrasoldatiLuis Marinas-PardoAndrea GalliLuis Mario Vaquero-RonceroAndrea GallinaLuis Miguel Sáez-AlcaideAndrea GarattiLuis MöckelAndrea GiacomelliLuís R RaposoAndrea GrilloLuis Rodriguez FrancoAndrea LechiancoleLuis Sánchez-GuillénAndrea MartiniLuis Suso-MartíAndrea MingoliLuisa AverdunkAndrea NegroLuisa BrussinoAndrea PantaloneLuisa FrizzieroAndrea PárniczkyLuisa LimongelliAndrea PassantinoLuisa María BotellaAndrea PhillipouLuisa SeoaneAndrea PoloLuisa Warchavchik HugerthAndrea RadinovicLuise HolzhauserAndrea RoccuzzoLuise WagnerAndrea RussoLukas B. BeenAndrea S. GrahamLukas FiedlerAndrea S. Mendoza-VasconezLukas JenneweinAndrea SalzanoLukas KoflerAndrea SambriLukas PeterAndrea SechiŁukasz CichockiAndrea SistiŁukasz DembińskiAndrea Stevenson WonŁukasz KruszynaAndrea TinelliŁukasz ŁapajAndrea TinnirelloŁukasz MazurkiewiczAndrea TognùŁukasz PałkaAndrea V. MargulisŁukasz SzczukowskiAndrea VescioŁukasz ZapałaAndrea Vukic DugacLuke J. NeyAndreas BleslLuke Y. ChenAndreas GoetteLut TamamAndreas HartmannLutz KonradAndreas JaegerLuuk OtterspoorAndreas KalogeropoulosLuzalba Sanoja-FloresAndreas KarakatsanisLydia J. FinneyAndreas KesslerLydia M. CastelliAndreas NeffLyndon KimAndreas NilssonLynn A. FussnerAndreas NüsslerLynn Mørch-JohnsenAndreas PüspökLynne BingleAndreas SchlatterLynne MillarAndreas SeitzM. Carmen Terol CanteroAndreas StengelM. Donald BlaufoxAndreas VychytilM. Paula MacedoAndreas WarnkeMaayan ShachamAndreas WidmerMacarena Gomez-LiraAndreas Wolfgang ReskeMacchi AlbertoAndrée Ann BarilMaciej FrantAndree KurniawanMaciej GawlikowskiAndrei V. KrassioukovMaciej J. KotowskiAndreia Martins RosaMaciej MachaczkaAndrej NowakowskiMaciej PolakAndrej PalaMaciej SosnowskiAndrej WagnerMaciej StaporAndreja Trojner-BregarMaciej SterlińskiAndrés AgudeloMaciej W. SochaAndres M. RubianoMaciej ŻukowskiAndrew BentleyMădălina-Gabriela IliescuAndrew ChanMaddalena Di SanzoAndrew Conway MorrisMadelynn ChanAndrew EnglandMadhavi GaviniAndrew GrannellMadhu OusephAndrew HopperMafalda SantosAndrew I. M. GreerMagali J. FontaineAndrew I. R. MaasMagdalena A. BryndzaAndrew Ian JoblingMagdalena DruszczynskaAndrew J. AndersonMagdalena DurlikAndrew J. GoldbergMagdalena KostkiewiczAndrew James ShapiroMagdalena Krajew-ska-WłodarczykAndrew JinMagdalena KrintusAndrew K. MartinMagdalena Kwiatosz-MucAndrew KingMagdalena Lukaszewska-KuskaAndrew LucasMagdalena Mackiewicz-MilewskaAndrew McKinstry-WuMagdalena WujtewiczAndrew RetterMagnus Bjorkavoll-BergsethAndrew S. DayMagnus SjogrenAndrew TubbsMagnus SvartengrenAndrew UklejaMaguy Del RioAndrew W. GillMahendra T. BhatiAndrew W. JoyceMahesh GajendranAndrew XanthopoulosMahesh IddawelaAndrew–Paul DeebMaheswari MuruganandamAndrey D. NasledovMaheswari SenthilAndrey KruglovMahima SinghAndrey V. DubovoyMahmoud DiabAndrey V. ZinchukMahmoud KallashAndrzej BieńkiewiczMahsa JavidAndrzej GłowniakMai RosenbergAndrzej L. ChamieniaMai Salah El Din Mohamed HelmyAndrzej M. KurylcioMairi PucciAndrzej MichalskiMaite Velázquez MartínAndrzej PrzybylskiMajdy IdreesAndrzej SilczukMakiko KumagaiAndrzej SkorekMakoto AokiAndrzej Za̧bekMakoto HashimotoAneta AleksovaMakoto HosoyaAneta Cymbaluk-PloskaMakoto MotomiyaAneta OlszewskaMakoto SuzukiAneta Ostróżka-CieślikMakoto TsujitaAneta R. BorkowskaMalek MakkiAneta Słabuszewska-JóźwiakMałgorzata Buksińska-LisikAnette Kjærbye-ThygesenMałgorzata ChlabiczÁngel Bernabé-GarcíaMałgorzata Dorota SkibinskaÁngel M. Soriano CastrejónMałgorzata Geszke-MoritzÁngel SevillanoMałgorzata JamkaAngela Dalia RicciMałgorzata KapralAngela Georgia CaticMalgorzata KolpaAngela H.E.M. MaasMalgorzata KonieczynskaAngela HorvathMałgorzata Kotula-BalakAngela M. CrawleyMałgorzata M. Tafil-KlaweAngela T. BiancoMalgorzata Muc-WierzgonAngelika IllgMałgorzata Opydo-ChanekAngelika Ludtke ErwinMałgorzata Pańczyk-TomaszewskaAngeliki AngelidiMałgorzata SobieszczańskaAngelina MeirelesMalgorzata WasniewskaAngello MinellaMalik AydinAngelo A. MarraMalin BrundinAngelo ArmandiMalin ErnbergAngelo BasterisMalte HolschenAngelo BellinviaMalte OhlmeierAngelo CastelloMalvin N. JanalAngelo Dell'AquilaMamoru KusakaAngelo M CarellaMamtha BallaAngelo PicardiManabu NatsumedaAngelo RuggieroManami KinjoAngelo SabagManasa K. NayakAngelo TroedhanManel LujànAngelo ZavattiniManel Sabaté TenasAngelo ZoliManesh Kumar Panner SelvamAngelo ZulloManfred HeckingAngelos HalarisManish Vijaya KumarAngelos KyriacouManjiri Kiran DigheAngels EscorsellMan-Jong LeeAngrit StachsMan-Kyo ChungAnibh M. DasManlio MilaneseAnica KlockarsManlio SantilliAniello MaieseManoj A. BiniwaleAnil Kumar JonnalagaddaManon BosAnirudh SharmaMansoor BangashAnish BahraMansoor M. NasimAnita Lyssek-BorońManuel Aguilar-DiosdadoAnita N. DattaManuel Alfredo PodestàAnja DiemManuel Barreiro-PérezAnja FlamezManuel BelliAnja GeislerManuel Carnero-AlcázarAnja Kathrin JaehneManuel Castro-CabezasAnja QuastManuel Coheña-JimenezAnja SchorkManuel Durán PovedaAnja Susanne MühlfeldManuel Gonzalez De La RosaAnjul KhadriaManuel MadrazoAnke C. FenderManuel Marques FerreiraAnke SchwarzManuel Martinez-SellesAnkit B. PatelManuel Menendez-GonzalezAnkit ManglaManuel NovalesAnkita GargManuel O. LagravèreAnkita ShuklaManuel Pabón-CarrascoAn-Kuo ChouManuel Pedro PereiraAnn A. VerhaegenManuel RequenaAnn BugejaManuel RichterAnn IgoeManuel Rodríguez-VallejoAnn M. NeumeyerManuel Rosa-GarridoAnna Arnal-GómezManuel SantamariaAnna BalatoManuel ZweckerAnna BednarekManuela Adelinde AschauerAnna BenrickManuela CampisiAnna BersanoManyoo A. AgarwalAnna BizońMara GavazzoniAnna BlasiMarc BernaAnna CamporesiMarc DeanAnna CieślińskaMarc FischlerAnna DanielewiczMarc HilhorstAnna E. LokshinMarc Olivier GauciAnna FabisiewiczMarc PawlitzkiAnna GoncéMarc ScherlingerAnna GranathMarc SimardAnna Kabłak-ZiembickaMarc Van Der ValkAnna KajdyMarc VivesAnna KlinkeMarcel LeviAnna KostopoulouMarceli ŁukaszewskiAnna KucharskaMarcella TazzariAnna LisowskaMarcella Van HoolwerffAnna Lis-ŚwiętyMarcello AugelloAnna LocatelliMarcello BosiAnna ManolopoulosMarcello CandelliAnna Maria FrustaciMarcello CherchiAnna Maria GianniniMarcello ChieppaAnna Maria PaolettiMarcello Francesco LinguaAnna Maria Siega-RizMarcello MaestriAnna Maria TestiMarcello ManfrediAnna Marie KupinskiMarcello MocciaAnna MattoutMarcelo CorreiaAnna Matysik-WozniakMarcelo De-Lima-OliveiraAnna MoniuszkoMarcin A JedrykaAnna MosiołekMarcin AdamczakAnna NowinskaMarcin FeldoAnna Olasińska-WiśniewskaMarcin HellmannAnna Olewicz-GawlikMarcin KozakiewiczAnna OliverasMarcin MaciejczykAnna PellatMarcin MilchertAnna PerriMarcin SadowskiAnna PisanoMarcin SamiecAnna Pokryszko-DraganMarcin SiwekAnna S. TzortziMarcin SochalAnna Serra RubertMarcin WełnickiAnna StaffasMarcin WłodarczykAnna StarzyńskaMarcin ZielińśkiAnna SurdackaMarco AllinoviAnna SuredaMarco BattistaAnna SzczegielniakMarco CascellaAnna SzpakowiczMarco CerranoAnna Szumera-CiećkiewiczMarco ChiostriAnna SzumilewiczMarco CintoniAnna TangMarco ConfalonieriAnna V. MaslennikovaMarco Conti NibaliAnna WajdaMarco DelsanteAnna WojciechowskaMarco FarronatoAnna WolskaMarco FiorentinoAnna ZaninoniMarco FiorilloAnnabel VreekerMarco GianiAnna-Karin WelmerMarco GiannelliAnnaleise R. Howard-JonesMarco InamaAnnalisa AnzaniMarco InvernizziAnnalisa BarbieriMarco MarencoAnnalisa BoscoloMarco Maria PascaleAnnalisa CarlucciMarco MendesAnnalisa Di CelloMarco MerliAnnalisa MilanoMarco MidullaAnnalisa PaceMarco MiglioratiAnnalisa TitoMarco MongilloAnna-Luisa KlotzMarco MontilloAnne Braae OlesenMarco MontorsiAnne E. KwitekMarco MorselliAnne E. VertiganMarco NardiAnne EkdahlMarco OderdaAnne FasslMarco PicichèAnne FlörckenMarco PignattiAnne HulinMarco PonzettiAnne K. GalgonMarco RoscignoAnne K. MühligMarco SchiavoneAnne KuusistoMarco SeracchianiAnne Margarette S. MaalloMarco SpilotrosAnne Nordrehaug AstromMarco TallaricoAnne Sophie BoureauMarco TorresAnne StrohbachMarco Van EijkAnne-Cécile PaepegaeyMarco Vito MarinoAnnekathryn GoodmanMarco WitkowskiAnnekatrin SteinhoffMarco ZanobiniAnnemarjut JääskeläinenMarco ZuinAnne-Sophie BergotMarcos Antonio Gonzá-lez-LópezAnne-Sophie MehdornMarcos PeriniAnnett SalzwedelMarcus BrooksAnnick PinaMarcus HackerAnnie MoulinMarcus HollenbachAnnie NarlaMarcus StoetzerAnnika AuranenMarek AsmanAnnika MalmströmMarek DedecjusAnn-Kathrin EichelmannMarek NalosAnoek A.E. De JoodeMaren GoeckenjanAnoop SheshadriMaren WeissAnselmo CaricatoMaresca Attard PizzutoAnthony AizerMargalida CalafatAnthony ArchibongMargaret JonesAnthony Barney HawthorneMargaret SherburnAnthony GerlachMargaret T. KasnerAnthony GuihurMargaret WalsheAnthony J. DixonMargarita Gozalo DelgadoAnthony LemaireMargaux Jenna KanisAnthony T. CorcoranMargherita BarbutiAntica MariastefaniaMargherita PizzicannellaAntoine AdenisMargherita SerraAntoine ChaanineMargit SerbanAntoine DrieuMargita PobijakovaAntoine E. SakrMarguerite VignonAntoine H. ChaanineMari C. Vázquez-BorregoAntoine LanotMaria A. BonifacioAnton R. KiselevMaria Adele GiamberardinoAnton StiftMaria Angela LosiAntonella Francesca Simo-nettiMaría Ángeles De MiquelAntonella GiordanoMaría Antonia Pastor-NietoAntonella ManniniMaria Arenas-MirasAntonella PiscioneriMaria BellringerAntonello D’AndreaMaria BloksgaardAntoni LluecaMaria BovaAntonia CamporealeMaria BreunAntonia Dimitrakopou-lou-StraussMaría Carmona‐IraguiAntonietta D’ErricoMaria Chiara MeucciAntonietta GiganteMaria ContaldoAntonija TadinMaria Da Luz CachuloAntonin TrimailleMaría Dolores BraquehaisAntonina I. FrolovaMaria E. MarketouAntonino DittoMaria Elena RiccioniAntonino ManiaciMaria Elisa MancusoAntonino MignanoMaría Eulàlia Fernán-dez-MontolíAntonino MorabitoMaría Eva Mingot-CastellanoAntonino NaroMaria Fernanda Sola RuizAntonino RubinoMaria Filomena BotelhoAntonio AntónMaria Francisca Colella-SantosAntonio AversaMaria FranziniAntonio BiondiMaria GazouliAntonio Casas-BarragánMaria Giulia NosottiAntonio CerasaMaría González-OlmoAntonio ChistoliniMaria Grazia DalfráAntonio ColantuoniMaria GuarinoAntonio CornoMaria HernandezAntonio De PascalisMaría Iglesias-GonzálezAntonio De VitaMaria IliadouAntonio FacciorussoMaria Irene BelliniAntonio Giampiero RussoMaria IsaguliantsAntonio GiorgioMaría José Bautista-LlamasAntonio Giuseppe BiondiMaría José García-PolaAntonio GrecoMaria José Yuste-SánchezAntonio J. AmorMaria KallergiAntonio J. Martin-GalianoMaria Kalliopi Konstan-tinidouAntonio LaghezzaMaria KitchenAntonio LanzaMaria L. MateusAntonio ManzelliMariá Librada Porri-ño-BustamanteAntonio Marcilla DíazMaria Lorena AbateAntonio Maria FeaMaria Lorenza MuiesanAntonio MarzolloMaria Luisa CalabròAntonio Mendes-SousaMaría M. Morales Suár-ez-VarelaAntonio MirijelloMaria Maddalena SirufoAntónio NogueiraMaria MajernikovaAntonio OrlacchioMaria Margherita RandoAntonio PalladinoMaria MazzitelliAntonio PontorieroMaría MedranoAntonio Popolo RubbioMaria MesuracaAntonio PretiMaria MilettaAntonio Ramos-MartinezMaria NyholmAntonio RomanelliMaría Orosia Lucha-LópezAntonio SalsanoMaria P. Martinez-CantarinAntonio SanzoMaria PaparoupaAntonio SchiattarellaMaría Pilar Pecci LloretAntonio SerranoMaría Prados-PrivadoAntonio Simone LaganàMaria Rita BiancoAntonio SiniscalchiMaría Roca LlorensAntonio Sitges SerraMaria RodriguezAntonio SorgenteMaria SeidelAntonio Tejera-VaquerizoMaria SkibińskaAntonio VozaMaria SofologiAntonio-Javier ChamorroMária SzekeresAntonio-Jesús Marín-PazMaria SzubertAntonios A. KoutalosMaría Teresa Arias-LosteAntonios ArgyrisMaria Teresa GandolfoAntonis FanouriakisMaria Teresa Gómez-CasaresAntonis SakellariosMaria Teresa TomasAntti LindgrenMaría Teresa Veláz-quez-MartínAnubhav KanwarMaria Therese AhlenAnupamaa J. SeshadriMaria TotanAoife EganMaria TsoliAparna ShindeMaria VittinghoffApostolos Georgiou PappasMaria Wieteska-MiłekApostolos GkatzionisMaría-Arántzazu Rues-cas-NicolauApril HerrityMariaconsiglia FerrieroAraceli García-MartínezMaria-Fernanda Lo-renzo-GómezArantza InfanteMarialucia GalloriniArash MotekallemiMariam Jamal-HanjaniAravind ThavamaniMarián Gómez-BeldarraínArgyrios IoannidisMariana TudoranAriadni SpyroglouMariangela ManciniAriane Laplante-LévesqueMarianna ArvanitakiArie Y.H. NemetMarianna CriscuoloArieh RazielMarianna K. TheodoraAriel Edward HightMarianna LeopoulouAriel FrajermanMarianna MeschiariAriel KorenMarianna RinaldiAriful IslamMarianne BrodmannArijit GhoshMarianne GwechenbergerArinc OzturkMariano CingolaniArindam BharadwazMariarita BrancaccioAris TsalouchosMariarosaria CampiseAristotelis PerrakisMariarosaria ValenteArjan VissinkMaricela C. ValerioArka ChatterjeeMarie Christine Morel-KoppArkadiusz LubasMarie Christine SimonArmando Coca RojoMarie DubarArmando Del PreteMarie EssigArmando L. OliverMarie G. GantzArmando Perez De PradoMarie Gisselsson-SolénArmando SenaMarie Hauguel-MoreauArmin KalenkaMarie Louise P. Van Der HoornArnaud BissonMarie N DelyferArndt StahlerMarie-Astrid Van DievoetArne Broch BrantsæterMarie-Christine VantyghemArne KandulskiMarieke J.H. BegemannArni S.R. Srinivasa RaoMariel G. KozbergArnold GeoffreyMarie-Louise BartelinkArnold H. SetoMarika TardellaArnon BentovimMariko HosozawaArnon BroidesMarilena GregoriniArnulfo Hernan Nava-ZavalaMarina Carrasco-LlatasAroa Tardáguila-GarcíaMarina FericArrigo CiceroMarina SciancaleporeArsalan ShahidMarina TrivisanoArthur TarriconeMarina Varela DuránArtur PasternakMarinka TwiltArtur WdowiakMarinos G. SotiropoulosArturo Blázquez-NavarroMario D ToroArturo CesaroMario De FeliceArun RajasekaranMario F. ScaglioniAsa Konradsson-GeukenMario ForcioneAsadolah MovahedanMario FruschelliAsaf AchironMario GanauAsaf D. YanirMario HevesiAsako MatsushimaMario Lozano-LozanoAsghar AliMario PacilliAsha TyagiMario Pérez-SayánsAshish J. MathewMario SalviAshish JainMario SenechalAshley Eleen MorganMario Wolfgang KramerAshley P. NgMariola Śliwińska-MossońAshwin PintoMarion MussbacherAsim EjazMarion RabantAsim KichlooMarion SchaeferAsmerom SengalMariona RiusAsmita MishraMarios RossidesAssaf BuchMarish OerlemansAstrid PinzanoMarisol Castillo-CastrejónAstrid WeissMarissa J. CarterAthanasia PapazafiropoulouMaristela Pinheiro FreireAthanasia PrintzaMarius HenriksenAthanasios BikasMariusz Aleksander BromkeAthanasios ChalkiasMariusz GoniewiczAthanasios D. GiannoukasMariusz HartmanAthanasios K. KonstantinidisMariusz KlenckiAthanasios PapathanasiouMariusz NiemczykAthanasios SaratziotisMarja L. LaineAthanasios VergadosMarjan Wouthuyzen-BakkerAthina Maria AloizouMark A. SamaanAthina PyrpasopoulouMark A SteinAtsuhiko YagishitaMárk Ádám AntalAtsuko NakayamaMark BlaserAtsuko ShonoMark ButlinAtsumasa KomoriMark DennisAtsunori ItagakiMark E. PepinAtsushi FukunagaMark F. SommerfeldtAtsushi KannoMark FitzgeraldAtsushi KohgaMark L. BrantlyAtsushi KohyamaMark LobbesAtsushi SakamotoMark M. WalshAtsushi SugiuraMark PraetoriusAtsushi TanakaMark R. De HoraAttila BokorMark Rami BarakatAttila FeherMark S. MandelcornAttila FrigyMark SwainAttila FrigyesiMark W. RoarkAttila JakabMark W. RussellAttila KovácsMarkku J. NissinenAude Sophie NassifMarko KonschakeAudrey AdjiMarko PopovicAudrey DelcenserieMarko ZdravkovićAudrius DulskasMarkus Alexander KüperAugusto Pietro CasaniMarkus Florian StevensAurelie PlessierMarkus J. AnkenbrandAurelien LouvrierMarkus LenzhoferAurélien VenaraMarkus MachAurelio QuesadaMarkus RihlAurelio Rodríguez-PérezMarkus SchleeAurora Castro-MendezMarkus W. HollmannAurora MaglioccaMarkus W LöfflerAurore BoutyMarkus WurmAvinash AujayebMarlena SchniederAvital HershkovitzMarlijn HoeksAviv WeinsteinMarrieth RubioAvner BelkinMarta Aguilar-PerezAxel BartoliMarta AlvesAxel SarriasMarta Araujo-CastroAya SaitoMarta BandeAyaho YoshinoMarta Carrillo-PalauAydin GülsesMarta Díaz-MartínezAysegül AksanMarta FichnaAyumu YamashitaMarta Garcia-ClementeAzadeh NazemiMarta KepinskaAzar RadfarMarta Matamala-GomezAzin ParsaMarta Nowakowska-KotasAzumi IshizakiMarta OpalińskaB. Anthony ArmsonMarta RachelB. Nirmal KumarMarta Ramon-KrauelBaba InusaMarta TononBabak E SaraviMarta VerneroBabatunde RajiMarta Waliszewska-ProsółBahil GhanimMarta Zaleska-KocieckaBal Krishna ChaubeMarta ZaràBala RamananMarten TrendelenburgBalazs NemethMartha KestlerBaldev Malkit SinghMartijn J.J. FinkenBaptiste LamarthéeMartin AltreutherBarak RosenzweigMartin Bjørn StausholmBarbara BaranowskaMartin BuechertBarbara BlundellMartin CourBarbara Bodner-AdlerMartin D. BergerBarbara CvenkelMartin EbingerBarbara DarišMartin H SteinbergBarbara De AngelisMartin John DedicoatBárbara GonzalezMartin Joseph BullockBarbara GordonMartin KlietzBarbara KabonMartin MullerBarbara Katharina GeistMartin Oliver SchmiadyBarbara M. JunghansMartin PienkowskiBarbara Mangweth-MatzekMartin Prince AlphonseBarbara RuaroMartin PunterBarbara RuggieroMartin Robert ChasenBarbara Ruszkowska-CiastekMartin S. MortensenBarbara S. WigginsMartin Sanchez-GomezBarbara SteinlechnerMartin ScharffenbergBarbara UlmMartin Schmidt-HieberBarbara YawnMartin SiepmannBarry McDonnellMartin TeraaBarry PaulMartin UrnerBart De GeestMartin WestermannBart PijlsMartin ZackBartłomiej PerekMartina BehanovaBartosz DalewskiMartina BonifaziBartosz KrzowskiMartina BuljacBartosz MalkiewiczMartina CebovaBartosz MiziołekMartina FerioliBartosz RymuzaMartina KroppBartosz SzmydMartina Maria MensiBartosz WojciukMartina NestiBashar HannawiMartina PezzulloBasil T. DarrasMartina RekatsinaBassam HallisMartino F PengoBayodé Roméo AdégbitèMarton AsztalosB.C. DannerMarvin MinkusBeat HintermannMarwan HabibaBeata GodlewskaMary Ann EmanueleBeata KaletaMary K. LynchBeata Kasztelan-SzczerbińskaMary P. McGowanBeata Matyjaszek-MatuszekMaryam ArdalanBeata OlejnickaMaryna BasalayBéatrice Brembilla-PerrotMarzena DębskaBeatrice OehlerMarzena Kurzawa-AkanbiBeau ToskichMarzena LaskowskaBecky D. ClarksonMarzenna ZielinskaBehnam Rezai-JahromiMarzi ChiaraBela FulesdiMarzia SegùBelén Alfonso-BartolozziMaša KandušerBelén García-MedranoMasafumi GotoBeloosesky RonMasaharu HataBen Arthur MarsonMasahiko NakataBen CarterMasahiko TsuchiyaBen CostelloMasahiro HiranoBen GibbisonMasahiro SeikeBen Willem MolMasahiro ShinodaBen Z KatzMasahiro YoshikawaBence CsapóMasakazu HirotaBenedetto IelpoMasakazu IshiiBenedetto TerraciniMasaki MiyasakaBenedict SeoMasaki OkamotoBénédicte NobileMasanao NakamuraBenedikt PreckelMasanori FujisawaBenedikt SchliemannMasanori KonoBenedikt SchwormMasanori MasuiBengi BaranMasanori TakeokaBengt NellgårdMasanori YoshizumiBeniamin GrabarekMasaru HonmaBeniamino TripodiMasaru YamaguchiBenito AlmiranteMasashi IshikawaBenjamin B. WilliamsMasashi NagaoBenjamin BenzonMasashi UeharaBenjamin D. ElderMasataka KikuyamaBenjamin DeniauMasataka SumiyoshiBenjamin EssayaghMasataka UmedaBenjamin HotteMasato MurakamiBenjamin MarchandotMasaya AkashiBenjamin NotoMasaya IwamuroBenjamin RaderMasayuki EndoBenjamin ShelleyMasayuki NagasawaBenjamin VoellgerMasayuki OtsukiBenoît BolandMasayuki OzakiBenoit GueryMasayuki ShinchiBente Thoft JensenMasayuki UrabeBen-Zion JoshuaMasoud AzodiBerangere S JolyMasrour MakaremiBerenice Caneiro-QueijaMassimiliano BeghiBernadeta SzewczykMassimiliano BonifacioBernadette CoricaMassimiliano CretaBernard CorenblumMassimiliano Del BeneBernard La ScolaMassimiliano GarzaroBernardete F. MeloMassimiliano LanzafameBernardo StutzMassimiliano MoscaBernd StadlingerMassimiliano RuscicaBernhard Christoph DannerMassimo BaraldoBernhard H.F. WeberMassimo BelliniBernhard RauchMassimo BolognesiBernhard WernlyMassimo ColomboBernhard ZelgerMassimo GalliBernward LauerMassimo ManginoBert AnderssonMassimo MartinoBert J. M. Van De HeijningMassimo MattioliBert Jan Potter Van LoonMassimo NapolitanoBert-Ram SahMassimo PrimignaniBertrand RozecMassimo TruccoBeth MallardMassoure Pierre-LaurentBetsy FoxmanMatei BratuBettina EngelMateusz CybulskiBettina GohlkeMateusz KowalBettina HohbergerMateusz Krystian HołdaBharani Krishna Mynampati ArunadithyaMateusz KuklaBhavisha A. PatelMateusz PusleckiBiagio BaroneMateusz RadwańskiBiagio CangianoMateusz SpałekBianca GrosserMateusz SpiewakBijar GhafouriMateusz WierdakBilal KhokharMateusz WiniarczykBiljana Popovic-TodorovicMateusz ZurowskiBiljana StankovicMathews VargheseBimmer ClaessenMathias BurgmaierBing HanMathias OrbanBirgit LangeMathias TrempBirgit MarkusMathias WoschekBirol TopçuMathieu BergerBjoern Christian LinkMathieu JozwiakBjörn SommerMathieu NacherBjörn StranderMathilde Body-MalapelBjörn TampeMathilde DaxheletBjörn WeissMatija BarbičBlanca De-la-Cruz-TorresMatija MilosevicBłażej CieślikMatisyahu ShulmanBłażej MęczekalskiMats C. RaneboBłazej MisiakMats ErikssonBo Young ChungMats SjöströmBoaz NachmiasMatteo BaucknehtBogdan CatargiMatteo BrucoliBogdan DorofteiMatteo ChiappediBogdan GhermanMatteo De PastenaBogdan SoceaMatteo FermiBogusław MikaszewskiMatteo GhilliBoguslawa LuzakMatteo GiovarelliBohdan WasilewskiMatteo LucchiniBojana BeovićMatteo Luigi Giuseppe LeoniBolesław KalickiMatteo MartinatoBoniphace KutelaMatteo MorettiBoohwi HongMatteo NardinBortolo MartiniMatteo NeriBorys M. KwintaMatteo PaganiniBote BruinsmaMatteo PagnesiBoudewijn E. C. PlaatMatteo PaolucciBoulos NassarMatteo PigaBourke W. TillmannMatteo RenzulliBożena Romanowska-DixonMatteo RiccòBożena Szyguła-JurkiewiczMatteo SchimberniBraid A. MacRaeMatteo SorgeBrandon A. MillerMatteo TacelliBrandon G. SmagloMatteo TebaldiBranka Petrić-MišeMatteo VirdisBraxton D NollMatthew C. CookBreda CushenMatthew G. BrewerBrendan C. StackMatthew J BartonBrent RaiteriMatthew J. FriedmanBrent WagnerMatthew J. HamiltonBrian A. MendelsohnMatthew JoseBrian BieberMatthew LeinungBrian Bridal LøgstrupMatthew Meng Yang LeeBrian CrookMatthew Q. MillerBrian D. LoMatthew T. StrattonBrian E. GrottkauMatthew ZammitBrian J. ClayMatthias BuechterBrian K. FoutchMatthias D. HoferBrian Lowell BadmanMatthias D. WimmerBrian WalittMatthias Daniel ZinkBridget StroupMatthias DerwallBrigida BarberioMatthias J. RoggendorfBrigitte HolzingerMatthias KarstBritta A. KuehlmannMatthias M. MauschitzBritton BrewerMatthias MillesiBrody FoyMatthias SchneiderBronisława Skrzep-PoloczekMatthias SchulzBruce F. ScharschmidtMatthias SteinertBruce H. CohenMatthias UnterhuberBruce MorlandMatthias WalterBruce RothschildMatthias WuttkeBrunelli StefanoMatthieu MillionBruno DonatucciMatthijs MoerlandBruno FalcomatàMatthijs Paul SomfordBruno FernandesMatti Iso-MustajärviBruno FiondaMattia BarbotBruno GeierMattia BusanaBruno LaviolleMattia ChiesaBruno LorenziMattia D’AgostinoBruno M. FonsecaMattia MorriBruno VogtMattia Russel PantaloneBryan WinnMattias HofmansBryan YoungMaura M. ZyllaBumhee YangMaureen HansonBurhan GharaibehMaurizio BottiroliBurra MadhukarMaurizio Cappelli BigazziByoung-eun YangMaurizio MarchesiniByron VaughnMaurizio MenichelliByung Yi KoMaurizio MiglinoByung-Kwan SeoMaurizio NordioC. Bulai LivideanuMauro BiffiC. LeiberMauro CancianC. Noel Bairey MerzMauro CeccantiCailin JordanMauro PepiCaio Victor SousaMauro PettorrusoCaitlin TakahashiMauro PoddaCaitriona M. McEvoyMax Carlos Ramírez-SotoCaleb J. KellyMax SchlaakCălin CăinapMaxime TouzotCalin PopaMaximilian BreuerCalogero Lino CiramiMaximilian BrunnerCalum D. MoultonMaximilian DeestCalvin EiberMaximilian DietrichCamelia BucsaMaximilian Franz Schul-ze-HagenCameron G. EstrichMaximilian SeidlCameron W. MacDonaldMayank KansalCamile FarahMayssam NehmeCamilla CalvieriMayur ChoudharyCamilla DrexlerM.D. ArguisuelasCamilla Kjellstad LarsenMd Daud Mohd KhairiCamilla S. GrahamMd. JakariaCamille A. ClareMd Khadem AliCamille TlemsaniMd MohiuddinCampbell H. ThompsonMeaghan CloughCan CuiMegan Christine Hethering-ton-RauthCan ÖztürkMegan L. UhelskiCandelaria VergaraMegan Lee NeelyCara E. Capitena YoungMegan SutterCarina DantasMegha AggarwalCarina Sparud LundinMeghan SebastianskiCarl Christoph GoetzkeMehdi OualhaCarl ErbMehdi SakkaCarl Mikael ÖbergMehdi TavakoliCarla ColomboMehmet DalkizCarla Costa LançaMehmet GülCarla De GiovanniMehmet KocaogluCarla PalmaMehmet M AltintasCarla StartinMehrdad BehniaCarles Díez-LópezMehrez ElnaggarCarliene Van DronkelaarMei Lan KoCarlo Alberto RicciardiMeir Mei-ZahavCarlo Andrea PivatoMeiyo TamaokaCarlo BastianelliMelanie A. GunawardeneCarlo BelliniMelanie L. PlinsingaCarlo BertoncelliMelanie WalkerCarlo BizMelda SaglamCarlo BudanoMelis N AnahtarCarlo CavaliereMelissa AmundsonCarlo GaniniMelissa MaczisCarlo M. OrangesMelissa Renée ChambersCarlo SaccardiMelvin F. LorenzoCarlo TicconiMengxi ShenCarlo TumscitzMercedes Rivas-LasarteCarlo VignatiMeredith GreshamCarlos AlvesMeritxell MunmanyCarlos Bernal-UtreraMervi KanervaCarlos CrastoMetis HasipekCarlos Cuenca-BarralesMette Nåmdal VesterhusCarlos E. VelascoMi Kyoung SongCarlos Eduardo Fonse-ca-AlvesMi Yang JeonCarlos López-de-CelisMiao HeCarlos Luque-MorenoMicaela De PaloCarlos M. CoelhoMicah ZuhlCarlos Medina-PerezMichael A.E. RamsayCarlos Miguel MartoMichael BatemanCarlos Navarro CuéllarMichael BerlinCarlos O’Connor-ReinaMichael ChoiCarlos RochaMichael C.Y. NamCarlos Rodriguez-LopeMichael CzihalCarlos Rodríguez-LozanoMichael D. PerloffCarlos Romero MoralesMichael D. SpartalisCarlos Vicario-AbejónMichael DieckmeyerCarlotta PipoloMichael DiepersCarlotta SpagnoliMichael DItilloCarme Santoyo-MedinaMichael DonahoeCarmelina C. ZirafaMichael F. YayacCarmelo BernabeuMichael FeichtingerCarmelo CalìMichael FrickerCarmelo GurnariMichael FuchsCarmelo Lo FaroMichael FurianCarmelo Massimiliano RaoMichael HanCarmen C. LlenaMichael HeneinCarmen CiavarellaMichaël HoferCarmen MannucciMichael HolzerCarmen RochMichael J. BrennerCarmen Rodríguez GarcíaMichael J. LipsonCarmen RoncalMichael J.A. TurnerCarmen Suárez-SerranoMichael JackaCarmina MontoliuMichael KlompasCarmine CitarelliMichael KnitschkeCarmine IzzoMichael L. BernardCarmine OstacoloMichael L. CherCarol ConradMichael L. KuehtCarole DeanMichael La FountaineCarolin V SchneiderMichaël LaurentCarolina Aguiar MoreiraMichael Lichtenauer,Carolina De CiuceisMichael M. NeekiCarolina NylenMichael MeyerCarolina SimioniMichael MickelCaroline ApraMichael MolitorCaroline E. ReinkeMichaël NaglerCaroline HoffmannMichael NekludovCaroline HundepoolMichael NorbergCaroline J. VranaMichael PaulussenCaroline JadlowiecMichael PfeilstöckerCaroline M. SpeksnijderMichael PinskyCaroline M. Van De HeyningMichael R. GoCaroline RoneyMichael R. JacobsCaroline VayneMichael RajnikCarolyn M. Smith-MorrisMichael RiedCarolyn SummerbellMichael SalnaCarsten KamphuesMichael ScharfschwerdtCarsten TjellMichael SchwaigerCaspar StephaniMichael Scott FreemarkCassandra E. HendersonMichael Seltz KristensenCassidy DelaneyMichael SpartalisCatalin TiliscanMichael StöckleCatalina FlorezMichael T. KoltzCatarina Duarte SantosMichael TannerCate MadillMichael TorzewskiCaterina AurilioMichael VeldemanCaterina FedeMichael VoulgarelisCaterina LannaMichael WaisbourdCaterina MancarellaMichael WorlicekCaterina TomaMichaela M. HellCaterina TramontiMichaela Veronika BonfertCatherine A ByrnesMichail DiakosavvasCatherine BirtMichail MavrosCatherine BissonMichał BoraczyńskiCatherine BungenerMichał BorysCatherine CroftsMichał ChyrchelCatherine McCartyMichał GinsztCatherine PenningtonMichał GontarzCatherine UzanMichal GranotCátia MagalhãesMichał Jan StasiowskiCécile BataillerMichal KovoCecile C De VosMichał KuszewskiCecilia ChaoMichal M. MasternakCecilia Matilde Fernández PonceMichal NowickiCecilia Pegelow HalvorsenMichał OczkowskiCecilia PerinMichal OrdakCecilia TettaMichał PopowCecilia VillalainMichał RabijewskiCédric LenormandMichal ShaniCedric ManlhiotMichalina BłażkiewiczCédric PastoretMichalis LiontosCees A. SwenneMichalis PanteliCees C. Van Den WijngaardMichel GalinskiCeleste DiasMichel MarreCelia Sánchez-RamosMichel PrudentCelina WojciechowskaMichel RoethlisbergerCéline ChapelleMichela IannoneCéline DeraisonMichela SabbatucciCésar Calvo-LoboMichelangelo LucianiCesar MorísMichele BoffanoCesare BaldiMichele CassettaCesare DanesinoMichele CompagnoCesare MassoneMichele CorrealeCesare TripolinoMichele D'AttilioCezar HonceriuMichele De MariaCezary WójcikMichele FigusChadwan Al YaghchiMichele FinottiChaeseong LimMichele FioreChahyun OhMichele KlainChai Hong RimMichele LanzaChaitanya GadepalliMichele LorenzoChakradhara Rao Up-pugunduriMichele MalaguarneraChamira RodrigoMichele MarinòChand Parvez Danka Mo-hammedMichele MazzolaChandana B. HerathMichele PortogheseChandni Maria JacobMichele RoccellaChandrakala Aluganti Nara-simhuluMichele S. RedellChang Hoon BaeMichele StoccheroChang-Hoon ChoiMichele VitaccaChang-Jun ZengMichelino Di RosaChangqing XieMichelle NisolleChantale LacelleMichelle PrasuhnChao GaoMichikazu NakaiChao-Hui YangMichinari HiedaCharalambos Panayiotou CharalambousMichinori MatsuoCharalampos SiristatidisMichio ItabashiCharalampos ThomasMicol RomanoCharles B. EatonMieszko WieckiewiczCharles C. CoddingtonMiguel Ángel CastellanosCharles CobbsMiguel Ángel Gallard-VigilCharles GuenanciaMiguel Ángel GoenagaCharles Hayfron-BenjaminMiguel Ángel Suárez-MuñozCharles Kyriakos VorkasMiguel Armengot-CarcellerCharles Van PraetMiguel Castelo-Branco SousaCharlotte DesprezMiguel EstravísCharlotte GenestetMiguel Malo-UrriésCharlotte Lund RasmussenMiguel RaimundoCharlotte SonigoMihaela MocanCharlotte SteenblockMihai Emil CăpîlnaCheal-wung HuhMihai StrachinaruChee Kay CheungMihály ÚjhelyiChelsie M. YoungMikael CohenChen-Chi WuMike T. JohnChenghung LinMikel San-JuliánCheng-Maw HoMikhail M. KostikCheng-Ming TsaoMikihiko KogoCheng-Tang ChiuMikk JürissonCheng-Wei LiuMikkel Fly KraghChenjie ShenMiklos BitayCheryl L. TranMiklos RohlaChia-Cheng LinMiklós TóthChiaki ToidaMikołaj DąbrowskiChiara BaianoMilan PiyaChiara BazzichettoMilan PrsaChiara BenedettoMilda RudzianskieneChiara ChieregoMilena DeptułaChiara DallagiovannaMilena ŚwitońskaChiara GiraudoMilica BorovcaninChiara LestuzziMiller MatthewChiara MaffeiMilos PjanicChiara MarroneMin JungChiara Maura CiniselliMina NashedChiara MinottiMina RhoChiara MoltrasioMing ChenChiara Paola ZoiaMinghan HuChiara PiraniMing-Horng TsaiChiara RomeiMing-Hsin YangChiara RossoMingyan HeiChiara RuoccoMinh Doan T. NguyenChiara SorberaMinhaj AlamChiara StoccoMin-Ho HanChiara SuffrittiMinhyung KimChiara TarantelliMinoo Khalkhali ZaviehChiara VeneroniMinoru HasegawaChia-shi WangMin-Woo LeeChia-Ti TsaiMin-Yen HsuChia-Yu ChiuMinyoung LeeChien-Hua HuangMinzhong YuChih Hung LoMiodrag FilipovicChih-Hsiang ChangMiquel Camafort-BabkowskiChih-Wei ChenMira M. MilasChih-Yu ChiMircea RivișChii-Min HwuMircea TampaChikako HaraMirco SchapherChi-Ming ChowMireia TondoChin-Chen ChuMireille L.A. Van GoethemChinenye R. DikeMireille LaforgeChintan GandhiMirela RaosChip HahnMiriam Cortes-CerisueloChisato ShimanoeMiriam EchevarriaChi-un ChoeMiriam FrenkenCho-Hao LeeMiriam GötteChris A. GentryMiriam MarksChris C. TangMiriam PikkemaatChris HL LimMiriam ZacchiaChris John PlummerMirian Santamaría-PeláezChrista BuechlerMirja TenhunenChristian Albrecht MayMirjam Gauri WinkelChristian BacciMirjam RenovanzChristian BergerMirjana IvanovićChristian CarulliMirko PretoChristian EhrnthallerMiroslaw BanasikChristian F. FreyschlagMiroslaw MarkiewiczChristian ForstMirto FolettoChristian GeltnerMirza PojskicChristian HakulinenMisa NakamuraChristian HannigMistuhisa TakatsukiChristian HappelMitchell CohenChristian IneichenMitsuharu OkutsuChristian KochMitsuhiro AokiChristian Lars PolteMitsumasa KishimotoChristian LückeMitsunori TadaChristian MackeMitsuteru NakamuraChristian Peter Giuliano HeinenMi-yeon EunChristian PhillipsMohamad GoldustChristian R. MorgensternMohamad SalkiniChristian ReepsMohamed AR SolimanChristian SohnsMohamed ElessawyChristian TanislavMohamed El-MeseryChristian WesemannMohamed I. ElsaidChristian WrobelMohamed M. AliChristian WunderMohamed M. DarwishChristiana KyvelidouMohamed MekhemarChristiana Raymond-PopeMohamed MutalibChristiane AlbrechtMohamed SalemChristiane SkåreMohammad AkbariChristilla Bachelot-LozaMohammad Al-AkcharChristin HeneinMohammad G. SaklayenChristina A. MelexopoulouMohammad H. JafariChristina JankoMohammad KhalifehChristina MejersjöMohammad Nazmul HasanChristine BraegelmannMohammad R. SaadatzadehChristine KeipertMohammad Reza AminiChristine Martinez-VinsonMohammad SaadChristine OlbjørnMohammad Younis HajeerChristine Perret GuillaumeMohammadreza ShalbafanChristine StierMohammed GhiboubChristine SwobodaMohammed GrawishChristo BesterMohammed ZiaeiChristodoulos E. Papado-poulosMohanraj SadasivamChristof AignerMohsen IbrahimChristof VulstekeMohsen MazidiChristoph B. WiedenrothMohsen Motie-ShiraziChristoph BorzikowskyMoiz VoraChristoph BuchtaMojca BizjakChristoph BührerMojca Zerjav TansekChristoph FaschingerMojtaba VaismoradiChristoph GranderMomcilo JankovicChristoph HiemkeMona VintilaChristoph MannMoneisha GokhaleChristoph MehrenMonica BerrondoChristoph R. BehemMonica BoirivantChristoph SinzMonica CurròChristoph SpangMonica De GaspariChristoph TappeinerMonica GanzinelliChristophe DuboisMonica HellesøyChristophe HeymesMonica LöfvanderChristophe MassetMonica MontagnaniChristophe OrssaudMonica Sorina LickerChristophe SzymanskiMoniek De MaatChristopher AllenMonika BudnikChristopher C YoungMonika E. RacChristopher D. KroenkeMonika FleckensteinChristopher DuCoinMonika GawałkoChristopher FangMonika GrabiaChristopher J WorsnopMonika KoziełChristopher Jen Lock KhorMonika KujdowiczChristopher KingMonika Lu-komska-SzymanskaChristopher KobierzyckiMonika M. GladkaChristopher L. JenksMonika M. PolczynskaChristopher M. MaulucciMonika Micha-lek-ZrabkowskaChristopher MarshallMonika PrendeckaChristopher Munoz-BendixMonika ProbstChristopher R. SnellMonika ScheerChristopher Seungkyu LeeMonika SzeligaChristopher StaleyMonika Szewczuk-BogusławskaChristopher V. HughesMonokesh Kumer SenChristopher WeyhMontserrat Garcia-GonzalezChristos BakirtzisMoo Kyun ParkChristos ChatzikyrkouMoo Yeol LeeChristos ChouaidMoo-hyun KimChristos FeloukidisMoonKi ChoiChristos K. KontosMorena ShkodraChristos K. YiannakopoulosMorgan BroggiChristos KontovounisiosMorisada HayakawaChristos RammosMoritz MeyerChristos V. RizosMorris BeshayChuanqi PengMorten C. MoeChuanyi M. LuMorten HesseChul Ho KimMoshe Rav AchaChul-hyun LimMotaz HamedChun YuanMotofumi SuzukiChun-An ChengMoumita DattaChunbo HeMoyo C KruytChung Sim LimMubarak MashrahChung-Ho E. LauMubin TarannumChung-Kan PengMudit MishraChungkwon YooMueez WaqarChung-Yao HsuMuhamad Alhaj MoustafaChun-Han LoMuhammad Ashfaq KhanChun-Hung SuMuhammad E. HaqueChun-Ming ChenMuhammad IrfanChun-Po YenMuhammad JanChunsen WuMuhammad KhurramChutima KunacheewaMuhammad Sohail KhanChyi-Feng JanMuhammad WaqasCindy BokobzaMuhammad ZafarCino BendinelliMuhammed GerçekCinzia FatiniMulazim HussainCinzia RotondoMulugeta Bayisa ChalaCiro CelsaMulyoto PangestuCiro IndolfiMurali Krishnan KolikondaCiro ManzoMutsuaki EdamaClaas H. HinzeMyeong-jun SongClaire De MoreuilMylène P. JansenClaire DossierMyoung-Ho HyunClaire DuflosMyriam RudazClaire DupuisMyron NevinsClaire GravesMyrto PapamentzelopoulouClaire J. FoldiMythili KalladkaClaire Nour Abou ChakraMyung Goo LeeClaire RigothierMyung-chul KimClara Benedetta ContiNabil EbraheimClara BonanadNabil MehtaClara CerratoNabil RashdanClara HjalmarssonNada AndelicClara Martinez-PerezNada LukkahataiClarence KhooNader M. HabashiClarissa Fantin CavarsanNadia AssantaClarissa M. LiuNadia LucaClas-Göran Af BjörkestenNadia MarascioClaude FranceschiNadia NajafiClaude L. CharvetNadia Sawicka-GutajClaudia Angela Di SilvestriNadja DornhoeferClaudia ArenaNady GolestanehClaudia Barros CoelhoNadya MorozovaClaudia Cecilia RestrepoNael AlakelClaudia CrimiNaftali SternClaudia CrociniNaga Venkata Sabarinath NeerukondaClaudia FilippiniNahid PunjaniClaudia Iveth Astudil-lo-GarcíaNaiara OzamizClaudia KedorNaichuan SuClaudia LeoniNaim Abu-FrehaClaudia MassarottiNaji KharoufClaudia PisanuNajib RahmanClaudio AndaloroNamhee KimClaudio CerchioneNamrata KhuranaClaudio IovinoNamrata SinghaniaClaudio LegnaniNan WuClaudio LuchiniNana ShinozakiClaudio MontaltoNanako YamadaClaudio TanaNancy DiazgranadosClaudio UrbaniNancy S. GreenClaudius SteffenNancy SolomonClaus PetersenNandini MoorthyClaus ZachariaeNandini NairClayton SwansonNan-Ping YangCleiton Augusto LibardiNaoaki SakataClément BarsNaoharu YagiClément MorgatNaoki AkamatsuClio RubinosNaoki AsanoClive KellyNaoki FujitaCoco C. H. De KoningNaoki FukudaCody B. JacksonNaoki IwamotoCody PeerNaoki NakayaColin DeaneNaoki SatoColin Gerard EganNaoki TajiriColin SteeleNaoki TanakaColleen Clarkin SchreyerNaoki YamadaColleen M. O’ConnellNaoko MatsuiColm A. Ó'moráinNaosuke KuraokaConcepción De-Hita-CantalejoNaotake FunamizuConcetta Di NoraNaoya MurashimaCongshan SunNapaporn TananuvatConrad FischerNarcís MasollerConsolato SergiNarihito NagoshiConstance L. MonittoNarimantas Evaldas Sama-laviciusConstantin T. LucaNarut PrasitlumkumConstantin Von SeeNastassia NavasiolavaConstantina AggeliNatalia E. Fares-OteroCoppola AndreaNatalia Mena-VázquezCorinna TrenkerNatalia RiveraCornelia BlumeNatalia RozwadowskaCornelia DeutschNatalia SzejkoCornelia DotzenrathNatalie Garzorz-StarkCornelis VosNatalie LefortCorrado BernasconiNatasa Vidovic ValentincicCorrado CampochiaroNathalie BoulleCorrado CrocettaNathalie DoorenweerdCorrado TagliatiNathan Alexander MoreauCosimo BruniNathan CorrellCosimo CollettaNathan MoreauCosme García GarcíaNathan W. CumminsCosta BachasNathaniel T. BerryCostantino ErraniNatividad BenitoCostantino LeonardoNatsu SasakiCostantino MancusiNatsuhiko EharaCostantino SchiaviNavaneeth Krishna Rajeeva PandianCostanza Maria CristianiNavarro-Gil MayteCostanza PellegriniNaveed MalekCourtney Anne FinlaysonNaveen KondruCourtney FitzhughNay Min HtunCourtney Gray MontgomeryNayra Anna Martin-KeyCourtney M. RowanNazario CarrabbaCraig R. BaileyNazario FoschiCraig Stephen WebsterNazario PortolaniCraig ThompsonNazia ChaudhuriCrescenzio SciosciaNeal W. FlemingCringu Antoniu IonescuNeelan PillayCristian BaicusNeeraj K SinghCristian DeanaNeeraj N. ShahCristian FrancavillaNefissa HammacheCristian Iulius SurcelNegar OmidakhshCristian MirvaldNeha NandaCristiana CalicetiNehal ElsherbinyCristiana CatenaNeil McGregorCristiano AmarelliNeil PortmanCristiano BalzanelliNejc PikoCristiano SpadaccioNele HerrefodsCristian-Radu JecanNele VandersickelCristina AntinozziNelly Schulz-WeidnerCristina BallaNemanja VujicCristina BaraleNerea AlmedaCristina Bilbao-SieyroNerea Méndez-BarberoCristina BostanNeriman ÖzkanCristina BotellaNeruban KumaranCristina BrancoNéstor VallecilloCristina CanovaNéstor Ventura-AbreuCristina CarvalhoNeville G. SuskinCristina Castro-AlonsoNevin HammamCristina CiucaNevo MargalitCristina DaiaNial QuineyCristina Eller-VainicherNiall M. H. McLeodCristina GalvanNiamh Lynam-LennonCristina GavriloviciNicasio Pérez-CastellanoCristina GluhovschiNiccolo RiccardiCristina Gómez-CasadoNicholas ChiuCristina LamasNicholas K. SchiltzCristina Ojeda-ThiesNicholas Rhys Me-djeral-ThomasCristina ScavoneNicholas WetterstenCristina SmolenschiNico NagelkerkeCristina TejeraNico PaganoCristina VassalleNicola A. ValenteCristina VilaplanaNicola AdamsCrnogorac Marija DjordjicNicola CarettaCsilla CzimbalmosNicola CosentinoCynthia M. TracyNicola De AngelisCyprian OlchowyNicola DecaroCyril EleftheriouNicola DöringCyril JacquotNicola MaffulliCyrus MotamedNicola MagnavitaD. Catherine WalkerNicola MontemurroD. GardinerNicola MumoliDacia Dalla LiberaNicola PavanDae Hyun YooNicola PetrosilloDag Henrik ReikvamNicola PuglieseDaichi SumiNicola Riccardo PuglieseDaisaku ToyoshimaNicola SquillaceDai-Soon KwakNicola StrenzkeDaisuke HondaNicola TartagliaDaisuke KatagiriNicola ZampieriDaisuke KikuchiNicolae GicaDaisuke NagasatoNicolae MironDaisuke SakaiNicolaos J. ChristodoulidesDaisy WilsonNicolas DelcourtDajana CuicchiNicolas FeltgenDale LangfordNicolas GiraudeauDale VimalachandranNicolas GirerdDalia KamelNicolas GirszynDalil HannaniNicolas HoertelDalila ScaturroNicolas J. DedyDamian P.C. MawerNicolas KahnDamian PiotrowskiNicolas KozakowskiDamian SkrypnikNicolas PaulDamiano MeninNicolas PierreDamiano PatronoNicolás Roberto Robles Pe-rez-MonteolivaDamiano RegazzoliNicolas SergeantDamien BonnardNicolas SöhlingDamien LanéelleNicolas ThompsonDamien LegerNicole Birgit ArweilerDamon P. EisenNicole Hauptman SiegelDan Dumitrascu-BirisNicole InnerhoferDan HabermanNicole M. TheodoropoulosDan M. DorobantuNicole PizzorniDan M. LivovskyNicoletta BigliaDan ZhaoNicoletta Di SimoneDana CramariucNicolò MartinelliDana MuinNidhi KapoorDaniel A. BaroneNidhish TiwariDaniel Arthur AlbertNiels Damkjær OlesenDaniel AugustineNiels KokotDaniel BarbosaNiels Kristian AagaardDaniel BraunNiels QvistDaniel C. MorrisNiels UldbjergDaniel Cabanillas-BalseraNiels Van Den BergDaniel ChristophNiels WeinholdDaniel CukorNigel GleesonDaniel DubinskiNihal BakeerDaniel EyraudNihar J. MehtaDaniel G.E. ThiemNikhil AwatadeDaniel GravesNikhil PaliwalDaniel HolzingerNikhil Suresh BhandarkarDaniel J. BeardNikhil VasdevDaniel KookNiki ChristouDaniel KronenbergNiki JurbergsDaniel KrowarschNiklas F. EhlDaniel L. HerrNiklas KrupkaDaniel Lee BourqueNiklas Thomas BaerleckenDaniel MongioviNiko KacirotiDaniel P. ZalewskiNikola GolenhofenDaniel PatschanNikolai AttardDaniel Pedregal-MalloNikolaos A. StavropoulosDaniel PoppNikolaos MachairasDaniel SchöttleNikolaos MagkasDaniel SidlerNikolaos Nikolaos SchizasDaniel ŚlęzakNikolaos PerakakisDaniel SoueryNikolaos PyrgidisDaniel StjepanovicNikolaos SiafakasDaniel Vasile BalabanNikolaos VlachogiannisDániel VéghNikoleta PrintzaDaniel VenaNikolina Jukić PeladićDaniël WolfNikos GorgoraptisDaniel WolffNikos NikolettosDaniela AmzarNikos ViazisDaniela CarmagnolaNilesh R. VasanDaniela CavacoNilkant S. PhadDaniela CevolaniNimesh A. PatelDaniela DamianiNina KelloDaniela GiulianiNing DingDaniela IvanšićNir BarDaniela MessineoNiranjan KhadkaDaniela MontiNirma D. PereraDaniela ParrinoNirvana SadaghianlooDaniela RegaNisha NairDaniela ViscontiNishant PuriDaniele AmparoreNiti GoelDaniele ArmocidaNoah Ray JohnsonDaniele BarbaroNoam Y. HarelDaniele CastellaniNobuaki MoriyamaDaniele FalsaperlaNobuaki ShimeDaniele FarsettiNobuhiko OkamotoDaniele Guerino BiasucciNobuhiro KikuchiDaniele LinardiNobuhiro TanabeDaniele MasaroneNobuhiro YodaDaniele MasseraNobuo TomizawaDaniele OrsoNobuto NakanishiDaniele PironiNobuyuki MurakamiDaniele SantiNobuyuki MurakoshiDaniele SantilliNolan Stuart HornerDaniele UrsoNor Azlida Mohd NorDanielle FribergNora DenglerDanijel SikicNora EisemannDanijela PiskulicNora KlötingDanila de VitoNora V. ButtaDanilo BuonsensoNorbert VeyDanilo MenichelliNorberto PericoDanuta GajewskaNoriaki NagaiDanuta Gąsior-PerczakNoriaki YoshidaDanuta Lietz-KijakNorihiko KamiokaDanuta Raj-KoziakNorihiro NishidaDanuta Rosolowska-HuszczNorio YamamotoDany JaffuelNorma B. OjedaDario BrunettiNorma G. Padilla-EguiluzDario CalafioreNuala MurphyDario MessenioNunes Farinha Carlos Ma-nuelDario PrunaNuno. Moura-CoelhoDario RuscianoNunzia PrencipeDariusz JuchnowiczNupur N. UppalDariusz KałkaNuria FarreDariusz M. LebensztejnNúria FarréDariusz OnichimowskiObada HasanDarko BožićObul Reddy BandapalliDarren LauOctavio CabaDarryn A. AtkinsonOded OhanaDasom KimOfir KorenDavid A. HollanderOfira ZlotoDavid A.J. WilsonOksana Anatolia ShlobinDavid Araújo-VilarOkyaz EminagaDavid Bar-OrOla Didrik SaugstadDavid BeversdorfOlaf BeckDavid BurdetteOle JungDavid C. GillespieOleg EpelbaumDavid CarballoOleg KaradutaDavid ChambersOleg PolonetskyDavid Changli WeiOleksa RewaDavid De La Rosa CarrilloOleksandr KonievDavid DonnermeyerOlga CiepielaDavid F. GaieskiOlga Giménez-PalopDavid ForsströmOlga KislitsinaDavid GrynspanOlga KruszelnickaDavid HoogewijsOlga Santesteban EcharriDavid J. AltschulOlga SeifertDavid J. BrinkmanOlga SukochevaDavid J. KopskyOlga Tura-CeideDavid J. RamseyOlha HalyabarDavid JaffeOliver AmbréeDavid K. BaillyOliver BeetzDavid KipgenOliver BrainDavid L. RowlandOliver C. ThammDavid LegouisOliver T. StirrupDavid M. ConradOlivia LeavyDavid M ManninoOlivier MalaiseDavid M. PoppersOluseye OgunmorotiDavid Mataix-ColsOmar AlkharabshehDavid MikhailOmar CauliDavid N. DanforthOmar Emiliano Apari-cio-TrejoDavid Noah SatherOmar M. ChehabDavid Noiva PerdigotoOmer EkerDavid NolanÖmer ElmaDavid O. IrvingOmri EmodiDavid OrlikowskiOndrej MestakDavid PagliaOnofrio LaselvaDavid PooleOnorina BerardicurtiDavid PoulsenOorvashi Roy PuliDavid RozekOren LapidDavid S. GoldfarbOren RomDavid S SergeevichevOriel SpiererDavid SalmanOriol Iborra-EgeaDavid SanterOrith Waisbourd-ZinmanDavid Sanz RubioOrnella PunzoDavid SchumacherOrsola Di MartinoDavid SinclairOsama El ShamyDavid SnowdonOsamu ManabeDavid StrosbergOscar Figueras-AlvarezDavid T. LiuOscar Jl MitchellDavid VandrouxÓscar Lado-BaleatoDavid Varillas-DelgadoOscar M CañeteDavid WestergaardOskar KornasiewiczDavid ZweikerOsvaldo PadillaDavide ArcanioloOthon PapadopoulosDavide BavaroOtto Michael LeschDavide Di LazzaroOury CécileDavide FilippiniOuti LinnarantaDavide Giuseppe RibaldoneOya AndacogluDavide MancinoØyvind S. BrulandDavide MedicaOzan DikilitasDavide MusuOzan Emre ErenDavide PataP. Mangala C.S. De SilvaDavide PisaniP. TrimpouDavide SartiniPablo BoixedaDavide ScozziPablo Cervera-GarviDavide ZellaPablo Guisado-VascoDavit L. AghayanPablo Jorge Marcos-PardoDavy PaapPablo Parra-MembrivesDawid SigorskiPachiappan ArjunanDawn J. CasterPaloma MassóDe Gaulle I ChigbuPaloma TruebaDean G. KaralisPamela BeachDean YimlamaiPamela MilitoDebanjan DasguptaPanagiotis DervenisDebnath MajiPanagiotis KorovessisDeborah McCahonPanagiotis PapoutsoglouDeborah S. JacobsPanagiotis PeitsidisDebra GookPanagiotis TheofilisDeepa A. MalieckalPanagiotis TsikourasDeepak ChandrasekharanPankaj AhluwaliaDeepak JoshiPankaj SinghDeepak Kumar KhajuriaPanteleimon E. Papakonstan-tinouDeepanshu JainPanupong HansrivijitDeepti AbbeyPaola AcetoDeidre HursePaola BianchiDeirdre De RanieriPaola BonavolontàDejan JakimovskiPaola FriedrichDeli LiuPaola GalozziDemetra AntimisiarisPaola MuggeoDemetrios ArvanitisPaola QuaresimaDenis BourgeoisPaola Sanchez-PeñaDenis E. NaumovPaolo AseniDenis EvoyPaolo BrusiniDenis GallotPaolo CameliDenis MonneretPaolo CampioniDenisa E FerastraoaruPaolo CasadioDennis OrgillPaolo CavorettoDennis RosenbergPaolo FavaDerek BoeldtPaolo FerrariDerek D. BerglundPaolo FormentiDerek McMahonPaolo Francesco ManiconeDer-Shin KePaolo LimoliDeshan F. SebaratnamPaolo MainoDesiree Nadine WusslerPaolo MancaDespoina ManousakiPaolo MarraDevin J. MillsPaolo MelilloDevis BenfaremoPaolo MeneguzzoDhanush HaspulaPaolo MonardoDhaval ChauhanPaolo NucciDhelal Al-RudainyPaolo P. PrandoniDhiraj AcharyaPaolo PaioniDhrubajyoti BandyopadhyayPaolo PalatiniDhyanesh A. PatelPaolo PalmaDiana CassiPaolo PeraDiana DmuchowskaPaolo PoggioDiana E. LakePaolo SpinnatoDiana ErnstPaolo TondiDiana L. MartinPaolo VincenziDiana L. SnyderPaolo ZamboniDiana MacKayParamita ChakrabortyDiana MartinsParaskevas GkolfakisDiana RamasauskaiteParichoy Pal ChoudhuryDianne StaffordParisa GazeraniDibyendu DuttaParth BhattDidier DuclouxParvis FarahmandDidier MutterPascal AlfonsiDiego FresegnaPascal BeuretDiego KaskiPascal De Santa BarbaraDiego MoriconiPascal ProbstDiego RaimondoPascale PaulDiego RibuffoPasquale AmbrosinoDiego Serrano-MuñozPasquale CapaccioDilip GarikipatiPasquale Di MaioDimitri M. DrekonjaPasquale EspositoDimitri PatrikiPasquale MastrorobertoDimitrios DegiannisPasquale ViolaDimitrios K. ChristodoulouPasqualino SirignanoDimitrios KaragiannakisPatil ShankargoudaDimitrios KorentzelosPatra CharalampakiDimitrios KotsirisPatrice MarquesDimitrios MagouliotisPatrice NaudDimitrios PanagopoulosPatricia Acosta-VargasDimitrios PatouliasPatrícia CarneiroDimitrios TziakasPatricia Doyle-BakerDimitrios VenetsanosPatricia Garcia-CanadillaDimitris LambrouPatricia GarnicaDimitry SayenkoPatricia HuelinDimy FluyauPatricia RodriguesDinesh K. ThekkinkattilPatricia Ruiz-LimónDinkantha KumararatnePatricia Switten NielsenDionysios TafiadisPatrick G. BradyDirk J. BoschPatrick Georg ZorowkaDirk SchädlerPatrick GoettiDirk TheegartenPatrick H. J. HemmerDirk Van De PuttePatrick M. GlassmanDmitri ShchekochikhinPatrick MaréchalDmitriy IvashchenkoPatrick R. WaldenDmitry SergeevPatrick TéouleDmitry TretiakowPatrik SchatzDohyung LimPatrik WennbergDolores Sánchez-RodríguezPatrizia BisiacchiDolors Rodríguez-PardoPatrizia FarruggiaDomenico AielloPatrizia FerroniDomenico AzzolinoPatrizia ManciniDomenico IovinoPatrizia PignattiDomenico MaisanoPatrizia ZaramellaDomenico PecoraPatrizio MazzoneDomenico PennaPatrycja KleczkowskaDomenico SiricoPatrycja KupnickaDomenico TamburrinoPatrycja LipińskaDomingo Pascual-FigalPatryk LipinskiDominic BertschiPaul B. KaplowitzDominick DePhilippisPaul BatchelorDominik PförringerPaul CarrierDominik PruskiPaul CollinsonDominik SamotijPaul D. SmithDominik SaulPaul E. StevensDominik StrzeleckiPaul G. O'DonnellDominika SkolmowskaPaul H. M. J. VermeerschDominique PriéPaul HahnDominique SchererPaul J CiclitiraDomizia VecchioPaul J. HansonDonald J. Weaver Jr.Paul J.A. HusbyDonald LobsienPaul JansonsDonatella CiarapicaPaul LesueurDonato D’AgostinoPaul M. GriffinDonato LiloiaPaul M. StewartDonato RigantePaul Michael HallerDöndü Üsküdar CansuPaul MonahanDong ChenPaul MozdziakDong Hee KohPaul N CardingDong Ho ParkPaul PirteaDong Wook JekarlPaul R.v. JohnsonDonghwan ParkPaul S. AmieuxDong-jin HwangPaul S. ThomasDongrong XuPaul StrongeDong-Seok GwakPaul SundaramDonna ZwasPaul Van SchilDorcas Obiri-YeboahPaula Fernández GarcíaDoreen E. ChungPaula IruzubietaDorin-Bogdan BorzaPaula Juiz-ValiñaDorit GamusPaula O'SheaDorota DymkowskaPaula PeixeDorota KaminskaPaulina DumnickaDorota Zarębska-MichalukPaulina FortunaDorottya K. De VriesPaulina MoscickaDouglas J. TottenPaulina Niedźwiedzka-RystwejDouglas P. BlackallPaulina WlasiukDouglas P MarxPaulino A. AlvarezDouglas RootPaulo FernandesDragutin IvanecPaulo Hilário Nascimento SaldivaDražan ButoracPaulo J. PalmaDrew KernPaulo MarcelinoDrew NassalPaulo Miguel PereiraDrosos Alexandros A.Pavana RottiDu Geon MoonPavel AntiperovitchDuarte MarquesPavel ZhabyeyevDuilio DivisiPavol BartikDumache Raluca-OanaPavol SzomolanyiDung-Tsa ChenPawan Kumar RautDuo ZhangPaweł BąkowskiDurdica MarosevicPaweł GaćDursun KırbaşPaweł KicińskiE. Sherwood BrownPaweł KleczyńskiEberhard GrambowPaweł KrukowEbonie RioPaweł Kubasiewicz-RossEckart HanekePaweł MatusikEdelle Field-FotePawel MielnikEdgar V. LermaPawel MiotlaEdison JahajPaweł NadziakiewiczEdit GaraPaweł ReichertEdith MonteagudoPaweł RobakEdmund TsePaweł WańkowiczEdoardo CipollettaPawinee RerknimitrEdoardo ManfrediPayam SadeghiEdoardo MarraniPedro ArmarioEdoardo PappaianniPedro BrugadaEdoardo RellaPedro CasadoEdouard GerbaudPedro Delgado-GuillenaEduard QuintanaPedro GilEduardo BossonePedro Gonzalez-MenendezEduardo D. GomesPedro Infante-CossioEduardo Flores-UmanzorPedro Moliner BorjaEduardo García-ReyPedro SerralheiroEduardo GutieŕrezPedro Silva CunhaEduardo Gutiérrez-AbejónPedro ZapaterEduardo Marques-CarneiroPegah AfraEduardo Ortiz-CruzPei-Chen LiEduardo PozoPeiheng GanEduardo TargaronaPei-Hsi ChouEdurne AlonsoPellegrino MazzoneEdward J. TestaPeng-Ju HuangEdward K. RodriguezPer LinqvistEdward KoifmanPer MagnussonEdward SauterPer WändellEdward WylegalaPerminder GulaniEdwin BölkePernille BøyesenEdyta BarnasPernille HolmagerEdyta ŁuszczkiPernille Tine JensenEdyta MądryPerrine BortolottiEdyta PiłkaPertti PirttiniemiEero LindholmPessia Friedman RubinEfrat SpiegelPetca Razvan-CosminEfrén Martínez-QuintanaPeter A LeeEfstathia K KapsogeorgouPeter AndrewsEfstratios GeorgakarakosPeter BaptistaEfthymios IliopoulosPeter BerlitEftychia DemeroutiPeter BottenbergEgidio BarbiPeter C. AmbeEgle KvedaraitePeter D. WestenskowEgle MiliaPeter EuEhsanul Hoque ApuPeter GänglerEhud ZigmondPeter GeorgantopoulosEiichi WatanabePeter GirmanEiji KashiwagiPeter H. SchurEinat Shalom-PazPeter HeeringEirikur SteingrimssonPeter HobsonEirini Kanella Panagiotopou-louPeter HouwelingEirini KyranaPeter John FriendEisa Tahmasbpour MarzouniPeter KorstenEisen LiangPeter KronenbergEitaro KodaniPeter LuntEjaz A. AnsariPeter M. AndersonEkaterina BaryshnikovaPeter MichielsenEkhtear HossainPeter MirtschinkEl Hadji M. DioumPeter NeliganElaine AronPeter ProdingerElaine WainwrightPeter ProffElbert AlmazanPeter R. LawrensonEleftherios AnastasopoulosPeter RossingElena BartoloniPeter RumneyElena BobescuPeter SanterElena BollettaPeter Schuff-WernerElena CampelloPéter TajtiElena GalliPeter Thye-RønnElena Lo PrestiPeter V. GiannoudisElena MarchioriPeter V. SmeetsElena Martínez-SanzPeter W. GrandjeanElena NetchiporoukPetra BüttnerElena RoccaPetros KarkosElena TarcaPetros MartirosianElena TombaPetros P. SfikakisElena-Mihaela CordeanuPhilip BielefeldEleni GeorgakopoulouPhilip EffraimEleni KletsiouPhilip GardinerEleni TheocharidouPhilip Georg FerstlEleonora CinelliPhilip HelliwellEleonora Dalla BellaPhilip K. McClureEleonora FaccioliPhilip MaesEleonora FavuzzaPhilip McClureEleonora GaetaniPhilip SarajlicEleonora M.C. TreccaPhilip-Christian NolteEleonora TerreniPhilipp BadorrekEleuterio Sanchez RomeroPhilipp BreitbartElham AfghaniPhilipp DammElham RezaeiPhilipp KriechlingEli D EhrenpreisPhilipp KrisaiEli MagenPhilipp LichteEli MansourPhilipp Paul LobmaierEliana CostanzoPhilippe BrouquiEliana MontanariPhilippe CollasElias DiarbakerliPhilippe CollotteElias KehagiasPhilippe CuvillonEliasz KańtochPhilippe D’AbadieElie H. MotulskyPhilippe E. DuboisElina LinetskyPhilippe GarrigueEline H. PloumenPhilippe GirardElio BignardiPhilippe GossetElio VenturiniPhilippe GuilpainElisa BelleiPhilippe LewalleElisa BelluzziPhilippe Matthias TschollElisa De PaolisPhilippe MeyerElisa GiacominiPhilippe OriotElisa Lopez-DoladoPhilippe R KoninckxElisa Martín-MerinoPhilippe R. MarteauElisa Sefora PierobonPhilippe RousselotElisabet GranstamPia Clara PafundiElisabete Cristina Nunes FernandesPia Titterud SundeElisabeth A. KapposPicó AlfonsoElisabeth LippertPier Luigi AntignaniElisabeth Nieveen Van DijkumPier Luigi StefànoElisabeth ReiserPier MannucciElisabeth Sornay-RenduPier Vitale NuzzoElisabetta CesaroniPierachille SantusElisabetta LiveraniPiercamillo ParodiElisabetta MoscarellaPierfrancesco PuglieseElisabetta PilottoPiergiorgio DanelliElisabetta PolizziPiergiorgio La RosaElisabetta TonetPiergiuseppe AgostoniElisabetta TosoPierluigi MeroniElisabetta ZanattaPierluigi ToniuttoElise LerouxPiero FarruggiaElise Van Der StouwePiero PapiElitsa DeliverskaPiero PolleselloEliya LevingerPiero Vincenzo LippolisEliza WasilewskaPierpaolo TrimboliElizabeth A EllinsPierre DuquetteElizabeth A. KayePierre MichelElizabeth A. StewartPierre SabouretElizabeth BruntPierre-Aurélien BeuriatElizabeth BuchbinderPierre-Emmanuel BouetElizabeth De KortPierre-Grégoire GuinotElizabeth HendrenPierre-Marie RoyElizabeth K. LundPieter DemetterElizabeth N. MadvaPieter W M BonnemaijerElizabeth UngerPietro BertiniElke GasthuysPietro CacialliElla GrilzPietro Emiliano DonedduEllen F. MoslethPietro IaffaldanoElmina Mammadova-BachPietro MorroneElmira JalilianPietro PellegrinoÉlodie SellierPietro PicernoEloisa RomanoPietro RuggieriElric ZweckPil Young JungElsa LorthePilar Alfageme-GarcíaElsebet OstergaardPilar NegredoElvire Pons-TostivintPilar Pérez-RosElzbieta MillerPil-sung YangElżbieta Pac-KożuchowskaPim PullensElżbieta PoniewierkaPinakin Gunvant DaveyElzbieta Zdankiewicz-ŚcigalaPinchas HalperinEmad IbrahimPinelopi ArvanitiEmad QayedPin-jung ChenEman A. HamadPin-Kuei FuEmanuel RaschiPin-Nan ChengEmanuela MinnaPiotr AlsterEmanuela PanniaPiotr DobrowolskiEmanuele CassioliPiotr DuchnowskiEmanuele ChisariPiotr GabryelEmanuele PignattiPiotr J. BachulEmanuele ScalaPiotr JanikEmely VerweyenPiotr K. KrajewskiEmiel F.M. WoutersPiotr KornetaEmil IsagulyanPiotr LeszczyńskiEmil RudolfPiotr Marian HoffmanEmil YlikallioPiotr MorasiewiczEmilia SawickaPiotr PoznańskiÉmilie ChalayerPiotr RolaEmilie Palmgren ColovPiotr RozentrytEmilio RodrigoPiotr WychowańskiEmilio VareaPiotr Z. SobanskiEmily BaumrinPipitsa N ValsamakiEmily CarlPippa BaileyEmily CoxPiyush BaindaraEmily Jane MacKayPoemlarp MekraksakitEmily L. SavocaPol Camps-RenomEmily P. McCannPo-Ling KuoEmily R. HajjarPooyan MakvandiEmily R. SpitzerPorin PerićEmily S.J. EdwardsPouya AlaghbandEmily WhitePrabhakar BastolaEmina TalakićPrabhavathi MaddineniEmir BencaPrabhudev Prasad Purudap-paEmma BarrettPradeep V. KadambiEmma D'IppolitoPrahlad HoEmma HamiltonPrakrati AcharyaEmma KempPranavkumar ShivakumarEmmanouil GiorgakisPranshu SahgalEmmanouil KapetanakisPrapa KanagaratnamEmmanouil V. DermitzakisPrasad SrikakulapuEmmanuel AndroulakisPratheepa RasiahEmmanuel FrimpongPreeti MalikEmmanuel MelloulPrem SomanEmmanuel Navarro-FloresPrithvi S. SankarEmmanuel NicolasPriya PrahaladEndrit ShahiniPriyanka KhareEnikő KovácsPriyanka SabharwalEnric I. CanelaProttoy HasanEnrica GiammarinaroPrytuła AgnieszkaEnrico AmmiratiPrzemysław PuzEnrico BroccoPui Man Rosalind LaiEnrico CarminaPui Wah HuiEnrico CerratoPuneet AgarwalEnrico CollantoniPuo-Hsien LeEnrico ConservaPuppo FrancescoEnrico GiordanPurushottam LamichhaneEnrico IaccinoPuvanalingam AyyaduraiEnrico MarinelliPuya Dehgani MobarakiEnrico PiccirilloQais RadaidehEnrique Berjano-ZanónQasim AlhadidiEnrique Gómez-BarrenaQian YuanEnrique Maraví-PomaQiangjun CaiEnrique RomeroQilai LongEoin MacCraithQiu Jiantai TimothyEra D. MikkonenQiuwang ZhangEraldo OcchettaQuirino CiampiEran HadarR. KocyłowskiEran MamanR. Taylor RipleyEray AtalayR. William CaldwellEren KurisRaanan MeyerEric A. SribnickRachael BaiducEric F. GrabowskiRachael BeswickEric LevesqueRachel KellyEric Nguyen-KhacRachel WestEric OlingerRadbod DarabiEric PasmantRadhakrishnan PanatalaEric P.F. ChowRadoslaw B. MaksymErica BuosoRadoslaw MlakErick SilvaRadu OlariuErik BeckmannRadzisław MierzyńskiErik BülowRafael BadenesErik EdströmRafael Casuso-PérezErik F. EriksenRafael DavalosErik KarlssonRafael De la Garza RamosErik SuRafael Escamilla-NunezErika AlboRafał Białynicki-BirulaErika Fernandez-VizarraRafał MorgaErika JoosRafał ObuchowiczErmete GiancipoliRafał PokrowieckiErnesto CrisafulliRafal WolnyErnesto JimenezRafal Z SlapaErsilia LucenteforteRaffaele AspideEsben CarlsenRaffaele GalieroEscriu CarlesRaffaele GiordanoEspen DietrichsRaffaele La PortaEstanislao Gutiérrez-SánchezRaffaele La RussaEsteban Sáez-GonzálezRaffaele LiuzziEstefania NuñezRaffaele NatellaEsterita AccogliRaffaele OrnelloEsther BrusseRaffaella CastagnolaEsther CuadradoRaffaella ColombattiEsther ErdeiRaffaella PanzaEsther Garcia-MoraleRaghavendra PalankarEsther García-MoralesRahmeh OthmanEsther LázaroRahul KumarEsther MaasRahul MallickEsther NistalRahul SanwlaniEstibaliz JarautaRahul ShekharEstíbaliz JiménezRahul VaidyaEszter Somogyi-GanssRahul Y. MahidaEthan AndersonRaimondas KubiliusEthan David CohenRainer SchmelzeisenEthan StrattanRaj J. ChovatiyaÉtienne KhouryRaja Chandra ChakinalaEtJan I. OlofssonRajagopal MuruganEtsuo ChiharaRajan SinghEtsuro BandoRajan VediappanEue-Keun ChoiRajat KapoorEugene KucharzRajendran JC BoseEugenia MatoRaji GanesanEugenia SanchezRajmohan DharmarajEugenio PedullàRalph WeberEugenio ProvenzanoRaluca Simona CostacheEujin ParkRaluca TomoaiaEun Jeong GongRamcharan Singh AngomEun Jeong ParkRamesh PeriasamyEun Ji LeeRami S. KantarEusebi ChinerRamit Maoz-SegalEuthymia VlachakiRamkumar KrishnamurthyEva Češ̈kováRamona SturmEva CulakovaRamya ShenoyEva Ekvall HanssonRan HarelEva KoetsierRan KornowskiEva LaerknerRan ZhangEva María Martínez-JiménezRandall J. RoperEva Martinez-JimenezRandy L. JensenEva MelerRanjit DeshpandeEva MezeyRanjithkumar RajendranEva OnjukkaRaphaël DuivenvoordenEva V. E. MadsenRaphael P. LuberEva W. IepsenRaphael R. BrunoEvaldo FaviRaphael Sanchez-SalasEvangelos MaziotisRaphael SchiffmannEvangelos MessarisRaphael WurmEvelien DirksRaquel AlvesEvelyn FinnemaRaquel BoiaEvelyn Jane MacdonaldRaquel Cantero-TéllezEvelyne D. TrottierRaquel CorripioEvelyne Lopez-CrapezRaquel Guillamat PratsEvgeny ErmakovRaquel L. BernardinoEwa BargRaquel López ReyesEwa DudzińskaRaquel Sebio GarcíaEwa GajewskaRares CraciunEwa KędzierskaRasa MladenovicEwa KluczewskaRasa ZarnegarEwa KoscielniakRashmi DeshmukhEwa MichalakRashmi GulatiEwa PochećRasmus GustafssonEwa SobolewskaRasmus S. TougaardEwa Straburzyńska-MigajRaúl Capote-PuenteEwa Więsik-SzewczykRaul Felipe Palma-AlvarezEwa WunschRaul Justo SanzEwa ZasadzkaRaúl López-IzquierdoEwan D FowlerRaul Torres-ClaramuntEwelina DziurkowskaRaveena RamphalEwelina Elert-DobkowskaRavi A. ThakkerEwelina Wawryk-GawdaRavi Philip RajkumarEwoud B. CompeerRavi S. TripathiF. BoixRavindra K. SharmaF. Javier Del RíoRavindra KumarFabia AttiliRay Vern MatthewsFabian BarbieriRazvan IonescuFabian HeroldRebecca ChenFabian J. BrunnerRebecca DiekmannFabian KnebelRebecca DislerFabián Pérez-GonzálezRebecca E. HoedemaFabian SommerRebecca E. StewartFabian TrillschRebecca Eli SadunFabian TroschelRebecca J. StoresFabiana AlleviRebecca Karp LeafFabiana LucàRebecca L. PaszkiewiczFabien BornertRebecca L. RossFabienne LangloisRebecca L. RuebnerFabio CampodonicoRebecca L. ThomasFabio Di DomenicoRebecca NantandaFabio GuoloRebecca PrattFábio Juner LanferdiniRebecca V. SteenaardFabio LeonelliRebeka SilvaFabio MaglittoRebekah StevensFabio MangiacapraRebekka MedertFabio PagellaRebolledo RolandoFabio ScarinciReece Andrew SophocleousFabio ZainaReem AlkilanyFabiola AtzeniReeta LamminpääFabrizio Canepa-EscaroReidar P. LystadFabrizio CondorelliReimer RiessenFabrizio LuppiReinhard KoppFabrizio SgolastraReinhard WindhagerFabrizio SorsReinhart SpeeckaertFadar Oliver OtiteRenae J. StefanettiFahd QadirRenata ChalasFahmi ShibliRenata VaraljaiFaidon-Marios LaskaratosRenato De-filippisFaisal F. HakeemRenato GalzioFangyao TangRenato GondarFani GkrozouRenato SeracchioliFanzani AlessandroRenato V. La RoccaFaranaz AtschekzeiRenaud TissierFaris F. BrkicRenaud VerdonFaris KhanRené HandschuFausto AssandriRené HartensuerFausto RigoRene Van EsFausto TucciRené WenzlFederica BarbagalloRenee GardnerFederica Dall'OglioRenee J. ChosedFederica Di BerardinoRenée ScheepersFederica Di ProfioReney HendersonFederica FilippiRen-Huei FuFederica FogacciRenzo BonofiglioFederica FurfaroResi PucciFederica MagagnoliRetnagowri RajandramFederica MoraniReuben Olugbenga AyelekeFederica PianiReza AbdiFederico A. GrassiReza AlizadehFederico CacciapuotiReza GholamiFederico CanaveseRhiannon V. McNeillFederico CorviRicard ValeroFederico FerrariRicardo Castro AlvesFederico FortuniRicardo Diaz MilianFederico GeraldiniRicardo LeãoFederico MarchesiRicardo Rebelo-GonçalvesFederico MeiRiccardo De CarlisFederico NalessoRiccardo GottardiFederico PieruzziRiccardo LenziFederico SeranaRiccardo ManfrediFederico ZicarelliRiccardo MasettiFedor LurieRiccardo MastroianniFedro Alessandro PeccatoriRiccardo MoiaFelice Eugenio AgròRiccardo NevolaFelice FemianoRiccardo VioFelice GragnanoRich KalletFelice SorrentinoRichard BrownFelicitas BucherRichard C. DowellFelipe AlconchelRichard DekhuijzenFelipe Coutinho Kullmann DuarteRichard FergusonFelipe KazmirczakRichard Gary BennettFelipe Vadillo-OrtegaRichard HummelFelix BendeRichard J. FantusFelix F. GirrbachRichard J. FishFelix GoeserRichard J. StarkFelix Javier Jiménez JiménezRichard J. StawellFélix Marcos TejedorRichard LassFelix ReschkeRichard N.W. HauerFelix RosenowRichard OwenFelix ScholkmannRichard P. G. Ten BroekFelix W. A. WaibelRichard PlaczekFelix WezelRichard SchwameisFeng-Ming TienRichard TrohmanFerdinand VogtRifat AhmedFerdinando PaternostroRiitta A. NiinimäkiFerdinando ToscanoRikke Krüger JensenFerdy Van GeestRima KregždytėFermín Fernández-CalderónRinaldo PellicanoFernanda Andrade OrsiRinat Gabbay-BenzivFernanda RochaRino FrisinaFernanda RodriguesRisheng XuFernando Capela E SilvaRishi Man ChughFernando Cartón-GarcíaRishi SharmaFernando CiveiraRisto A. KauppinenFernando DominguezRita ChiaramonteFernando FonsecaRita ConigliaroFernando LopezRita MencucciFernando MazzilliRita PavasiniFernando Ruiz SantiagoRitu MograFernando UrcolaRiyaz KabaFernando Vázquez-AlonsoR.J. SwijnenburgFerruh ArtuncRoald Flesland HavreFeyza F. DarendelilerRobert BuckiFiammetta SoggiuRobert EgermanFilemon S. Dela CruzRobert F. BentleyFilip De SomerRobert FlisiakFilip JansåkerRobert FrithiofFilip RaciborskiRobert G. BestFilipa CordeiroRobert GajdaFilipe Vilas BoasRobert GałązkowskiFilippo AgnesiRobert GoldinFilippo AucellaRobert HallifaxFilippo BertozziRobert J. HomerFilippo Della RoccaRobert KissFilippo GhinRobert L Giuntoli IIFilippo MaselliRobert P. LiddellFilippo NiccolaiRobert RobinsonFilippo PelizzaroRobert S. KassFilippo PiacentinoRobert SchleipFilippo PieralliRobert SeyfertFilippo TorielloRobert SkomroFilippo VittadiniRobert ŚlusarzFilippos TriposkiadisRobert SneydFilippos VingopoulosRobert StansburyFilomena De NigrisRobert StawskiFinbarr MartinRobert T. MeansFinny Monickaraj SwamidossRobert W. W. BiedermanFiona E.J. McDonaldRobert ZymlinskiFiona LimanaqiRoberta A BerardFiona ReidRoberta AngelicoFiona UlphRoberta AntonelliFiorentina AscenzioniRoberta BgeginskiFiras Abu AkarRoberta D'AmbrosioFlavia GhellerRoberta ForlanoFlavia NeriRoberta VenturellaFlavia V. GouveiaRoberto AbundoFlorence BoissierRoberto AiolfiFlorence BoitrelleRoberto AufieriFlorence LaiRoberto BianchiFlorencio Jr ArceRoberto BiniFlorent GobertRoberto CannellaFlorian BaillyRoberto CantelloFlorian GrahammerRoberto CasconeFlorian HuemerRoberto ChimenzFlorian ReckerRoberto CirocchiFlorian SeyfriedRoberto CocchiFlorin Ioan ElecRoberto ColasantiFlorin OnisorRoberto CuomoFlorin TriponRoberto FilippiFloris WuytsRoberto H. ParadaForrest BakerRoberto La RoccaFortunato LonardoRoberto LatiniFotios DrakopanagiotakisRoberto MadedduFranca DipaolaRoberto MaglieFranca GenestRoberto MantovanFranca NataleRoberto PeltriniFranca TaniRoberto PiroFrances Meier-GibbonsRoberto RonaFrancesc BalaguerRoberto ScicaliFrancesc MoresoRoberto Secades-VillaFrancesca AmbrosiRobin ChungFrancesca Boscolo SesilloRobin GoodwinFrancesca BuonomoRobin J. TyackeFrancesca BursiRocco AicaleFrancesca Di SoleRocco PapaliaFrancesca GainoRocco SpagnuoloFrancesca GimiglianoRocio Barrios-RodríguezFrancesca GioiaRocío De Diego CorderoFrancesca IacobellisRocio Eiros BachillerFrancesca MencarelliRocio ToroFrancesca Romana FuscoRodolfo Gabriel GattoFrancesca Romana RipaniRodolfo Gómez BahamondeFrancesca Romana SpinelliRodolfo IppolitiFrancesca SalaniRodrigo Jiménez GarciaFrancesca ToiaRodrigo Torres-CastroFrancesca VincitorioRodrigo Valdes-RodriguezFrancesca ZaraRogelio Gónzalez-GónzalezFrancesco AcerbiRoger E. G. SchutgensFrancesco AgostiniRoger L. RoysterFrancesco AlessandriRohit GulatiFrancesco AmatiRohit RanganathFrancesco Antonio SalzanoRoland KocijanFrancesco BellinatoRolf Alexander JanosiFrancesco BennardoRomain BourcierFrancesco BiancoRomain CapouladeFrancesco CannataRomain DidierFrancesco ChianconeRoman HaberlFrancesco CiodaroRoman LeischikFrancesco ClapsRomán MorenoFrancesco ColucciRoman NowickiFrancesco CoreaRomana StehlikFrancesco CucciaRomualdo Del BuonoFrancesco De ChiaraRon SaulnierFrancesco De FrancescoRon ShapiroFrancesco Di GennaroRonald B. BrownFrancesco Di MarioRonald JackupsFrancesco DonatelliRonald P. DeMatteoFrancesco FacchinettiRonald SrokaFrancesco FedeleRonan ZimmermannFrancesco FerriniRonen PerezFrancesco FontanaRonen Robert LekerFrancesco ForforiRong-Sen YangFrancesco FormicaRonit AbirFrancesco GallettiRonpichai Chokesuwat-tanaskulFrancesco GarzottoRoopma WadhwaFrancesco GaziaRosa AndiasFrancesco GigantiRosa Calvo-EscalonaFrancesco GossettiRosa LaurettaFrancesco GrippoRosa MichaelisFrancesco GuzziRosa Romero-MorenoFrancesco M. Quaranta-LeoniRosalia PaternoFrancesco MaestriRosalia RagusaFrancesco MarongiuRosalin MishraFrancesco MuratoreRosalind E. JenkinsFrancesco OlivaRosalyn SingletonFrancesco OrziRosanna DammaccoFrancesco PataRosario DonatoFrancesco PellegriniRosario González-MuñizFrancesco PellicciaRosemary LimFrancesco PetrellaRosenkran Sina CathérineFrancesco PetrilloRosetta RagusaFrancesco PradaRosnawati YahyaFrancesco ProcaccioRoss LeightonFrancesco RicottaRoss SummerFrancesco SaglioRossana BerardiFrancesco SaladiniRossella CacciolaFrancesco SantoroRossella PalmaFrancesco SartiniRossella SiligatoFrancesco SecchiRoxana PopescuFrancesco SelvaggiRoxane CompagnonFrancesco StilloRoxanne BavarianFrancesco StomeoRoy KessousFrancesco TambaroRoy MashiachFrancesco TartagliaRu LiFrancesco TondoloRuben Aquino-MartinezFrancesco TorellaRubén Cuesta-BarriusoFrancesco VerdeRubén Herrán-MongeFrancesco ZarantonelloRubén QueiroFrancisco A. Gómez-VeigaRubén Trigueros RamosFrancisco Álvarez-LermaRubina ShaikhFrancisco BautistaRuchi ShahFrancisco BuitragoRüdiger J. SeitzFrancisco DasíRüdiger Von Eisenhart-RotheFrancisco Do ValeRudolf BenzFrancisco Eugenio Cal-vo-IglesiasRuhul AminFrancisco Guillen-GrimaRui GasparFrancisco Guillén-GrimaRui SongFrancisco Herrera-GómezRuifeng HuFrancisco Javier Álva-ro-AfonsoRuksana HudaFrancisco Javier AscasoRümeyza Turan KazancıoğluFrancisco Javier Cano-GarcíaRuna LindblomFrancisco Javier Gar-cía-MartínezRune JohansenFrancisco Javier Povedano-MonteroRupert JonesFrancisco José HidalgoRuslan B. AlikhanovFrancisco MartínezRussel BurgeFrancisco MesaRussell F WarrenFrancisco Moscoso CostaRut Lucas-DomínguezFrancisco Murillo-CabezasRūta VaičiūnienėFrancisco NavarreteRute PereiraFrancisco Nieto EscámezRutger MatthesFrancisco TustumiRuth A. BensonFrancisco Vasques-NóvoaRuth A. SwanwickFrancisco Wilker Mustafa Gomes MunizRuth KaufmannFranck Farzad RahaghiRuth ZaslanskyFrancky Teddy EndombaRuwen BöhmFranco BassettoRyan J. AveryFranco GranellaRyan Jin Young KimFranco LacconeRyan M. PedrigiFranco LaghiRyan P. BradyFranco PepeRyan W. DobbsFrancois BagateRyo MatsuuraFrançois MargueritteRyo NakamaruFrançois MilordRyohei KuwatsuruFrançois MoisanRyohei TakadaFrançoise Borson-ChazotRyohei TakeuchiFrançoise Galateau SalleRyoma MichishitaFrank BesagRyosuke TonozukaFrank C. KoRyszard BraczkowskiFrank F. TuRyszard PiotrowiczFrank GiblinRyszard TomaszewskiFrank HaoRyu MatsuokaFrank Martin HaeckerRyuji SakakibaraFrank TackeRyunosuke HakutaFranka MessnerRyusuke HoriFrank-Peter TillmannS. Bobo TannerFrans H. C. De JonghS. Priya NarayananFrantišek DráfiS. ShepherdFrantisek SaudekSa Ra LeeFranz Josef SeibertSabin J. BozsoFranziska C. TrudzinskiSabina GaliniakFranziska DornSabina PodlewskaFrédéric BouissetSabine K. MaschkeFrederic CapelSabine ZittaFrédéric HameurySabino LuzziFrédéric RocheSabrina NordinFrederic SchlemmerSabrina Rahman ArchieFrederic StaermanSaburo MatsubaraFrederic TriponezSabyasachi MoulikFrederick H. SilverSachin MohanFrederik RaiskupSachio FushidaFrederike KramerSachio TakenoFrederike T. FellendorfSachio TsuchidaFredric D. GordonSadia AfrinFreya DroegeSadiya ShaikhFrisullo GiovanniSaeideh NozohouriFrits H.M. Van OschSae-Yeon WonFuhito HojoSaffiatou DarboeFulvio BorellaSahadev A. ShankarappaFulvio ZulloSahar EsfandyariFumi MasudaSahar HoushdaranFumiaki ShimizuSahar KassemFumihiro OgawaSahar S. AbdelmoneimFumio ChikamoriSahrai SaeedFunda TuzunSaifudeen IsmaelGabriel AltitSaiju JacobGabriel Claudiu TenderSaiko SugiuraGabriel DjedovicSajjad ShiraziGabriel Navarrete-VazquezSake J. De VlasGabriel SandblomSaldiva Paulo Hilário Nasci-mentoGabriela GarciaSalma TahaGabriela GarcíaSalomone Di SaverioGabriele CentiniSalvador García-DelpechGabriele FeniniSalvatore BattagliaGabriele Gallo AfflittoSalvatore BocchieriGabriele MelegariSalvatore CocuzzaGabriele NibbioSalvatore GiordanoGabriele NicoliniSalvatore PastaGabriele PozzatoSalvatore PianoGabriele SavioliSalvatrice MancusoGabriele SicilianoSalwa El TawilGabriele SimoniniSamanta MazzettiGabriele TumminelloSamantha AbramGabriella CsíkSamantha C. PattonGabriella DvorakSamantha K. RowbothamGabriella FabbrociniSamantha LeeGael B MorrowSamantha R. WeaverGaetan GavazziSamara RiceGaetano AlfanoSameh AttiaGaetano Antonio LanzaSami AkbulutGaetano AurilioSami TetriGaetano GalloSamir V. ParikhGaetano MaffongelliSamuel A. BerkmanGaetano RiemmaSamuel AsanadGaetano SantulliSamuel K. CamposGaetano ValentiSamuel N HeymanGagan FloraSamuel P. ScottGagan KumarSamuel R. FernandesGaia SampognaSamuel X. ShiGali MaorSamuele VaccariGalit WeinsteinSamy LachkarGamaleldin OsmanSamy M. RiadGamze BildikSamy MetyasGani BajraktariSana GhaznaviGaowa BaiSanda Mihaela PopescuGareth A. NyeSándor GyörkeGareth J. WaringSandra AmadoGarifallia SakellariouSandra Bašić-KindaGarima DwivediSandra CurinierGarrett BurnettSandra GavinhaGary D. SteinbergSandra GuidiGary FrancisSandra Jiménez-Del-BarrioGary H. ChangSandra LealGary J. FarkasSandra M. Martin GuerreroGary MorganSandra RojasGary S. MintzSandra Rovira-AmigoGasparini PatriziaSandra Santos-MartinezGaston J. H. FranssenSandra TammGaston J. PiñeiroSandro ArdizzoneGaurav JoshiSandro GianniniGaurav KumarSandro RengoGautham YepuriSandy ShultzGauthier RemicheSang Ho KwakGavin C.A. WoodSang Hoon KimGavin J. JohnsonSang Hun KimGayane MartirosianSang Hun LeeGazanfar RahmathullaSang J. LeeGebhard MathisSangah HanGediminas GaidulisSanggu LeeGeert DomSangita P. PatelGema FrühbeckSanja DespotovićGema MedinaSanja HromišGema Mendez-LagaresSanja IvkovicGema MiñanaSanja KezicGemma Caterina Maria RossiSanja Zoričić CvekGemma MarcucciSanjeet Singh Avtaar SinghGemma PenfordSanjeev MadaanGemma PratSanjeev ShuklaGengo SunagawaSanna MeriläinenGenis CardonaSanne J. GordijnGennaro Mariano LenatoSantiago BonanadGennaro RuggieroSantiago Jiménez-MarreroGeoffrey E. RoseSantiago NavarroGeoffrey EmersonSanto Raffaele MercuriGeoffrey ToflerSara AlexanianGeorg A. BöhmigSara De MartinGeorg B. EhretSara GalimbertiGeorg GelbeneggerSara Guerrero-AspizuaGeorg HalbeisenSara L. KinterGeorg JohnenSara LippaGeorge AthanassopoulosSara MonteiroGeorge BertsiasSara O'CurryGeorge DimitriadisSara PerettiGeorge FragoulisSara RattottiGeorge GoshuaSara RegnérGeorge KuoSara RicciardiGeorge MaitiSara Salehi HammerstadGeorge NotasSara ShimoniGeorge PadosSarah AnnesleyGeorge TavartkiladzeSarah BärGeorge TsaknisSarah FriedrichGeorge UmemotoSarah K. HoltGeorge ValasoulisSarah L. HarrisGeorges JourdiSarah M. JurickGeorgi I. WassilewSarah MartaindaleGeorgi VassilevSarah MichielsGeorgia KaiafaSarah PriorGeorgia KarpathiouSarah ShalabyGeorgia TrakadaSarah ThomisGeorgiana ConstantinescuSarah Trager RidgeGeorgios ChatzisSara-Joan Pinto-SietsmaGeorgios D. PanosSaranya PalaniswamyGeorgios DimitriadisSaraschandra Vallabha-josyulaGeorgios E. PapadakisSarath VijayakumarGeorgios LabirisSarbajit MukherjeeGeorgios ManousakisSardar M.Zia UddinGeorgios MourtzinisSareh ZhandGeorgios NarosSarel HalachmiGeorgios SchoretsanitisSaša FrankGeorgios TagarakisSasa RajsicGeorgios TheocharidisSasan MoghimiGeorgios TsivgoulisSascha TreskatschGeorgios VrakasSascha ZuberGeorgy Th. GuriaSasha Z. PriscoGerald ChiSaskia WandGerald FerrettiSatadru LahiriGerald HoltmannSathi A.V. DeviGerald JordanSathishkumar RamalingamGerald KrennmairSatimai AniwanGerald R. MarxSatoru EbiharaGérald RaverotSatoru KaseGerald SoffSatoru S. KudoseGerald ZernigSatoru TsujiiGerami D. SeitzmanSatoshi EbataGerasimos BaltsaviasSatoshi MitaraiGerhard CvirnSatoshi NaganoGerhard GarhöferSatoshi OnoGerhard HesseSatoshi TakahashiGerhard LitscherSatoshi WashinoGerhard SteinbeckSatoshi YamaguchiGeri KeaneSatoshi YuguchiGermain HonvoSatu KalliolaGermana De NucciSatyavani KaliamurthiGernot KriegshauserSaul FrenkielGerold BesserSaulo Gabriel Moreira FalciGerry CoghlanSaum B. GhodoussipourGerulf RiegerSaurabh ChawlaGerwin Alexander BernhardtSaurabh RajpalGholamreza DaneshSavalan Ba-bapoor-FarrokhranGiacinto TrioloSavio JohnGiacomo BaimaSavvas LampridisGiacomo BertazzoniSchlossman DeborahGiacomo FarìScott AnthonyGiacomo GaribottoScott D. MistGiacomo GiacaloneScott Derek BrownGiacomo M. VianiScott MatsonGiacomo MarchiScott TomarGiacomo MiglionicoSéamas WeechGiacomo OteriSean AvedissianGiacomo ZaccheriniSean Chun Chang ChenGiada MinelliSean O'NeillGiampiero ParrinelloSean RudnickGiampietro FarronatoSebastian BohnGian Andrea PelliccioniSebastian BöttgerGian Luigi CanataSebastian D. SchaeferGian Luigi GigliSebastian EulerGian Luigi NicolosiSebastian FerriGian Marco PoddaSebastian FindekleeGian Maria GaleazziSebastian GehmertGiancarlo ComiSebastian HörberGianenrico SennaSebastian KrugGianfranco CalogiuriSebastian MajewskiGianina LucaSebastian PonsGianluca AimarettiSebastian SchadeGianluca BagnatoSebastian SchnaubeltGianluca CaiazzoSebastiano A. G. LavaGianluca CampoSebastiano CiccoGianluca CoppolaSébastien BommartGianluca EspositoSebastien HascoetGianluca FranceschiniSebastien JacquelinGianluca ManniSébastien MolièreGianluca PiatelliSébastien SchmerberGianluca R. DamianiSeçil VuralGianluca Raffaello DamianiSecondo FolliGianluca RigatelliSecundino FernandezGianluca RompianesiSeigo NishidaGianluca SampietroSeigo OhbaGianluca SerafiniSeiichi OkabeGianluigi PastaSeika DenGianmarco AbbadessaSeishi MurakamiGianmaria D'AddazioSeisuke OkazawaGianmaria SalvioSelcuk ErdemGianni Di GiorgioSelcuk KeskinGianpaolo VaccarellaSelene BaroneGil D. HoftmanSelene Pérez-GarcíaGilad YahalomSema G. QuadirGilbert HangelSémah TagouguiGilberto A. GonzalezSemra BilacerogluGilles Barone-RochetteSengchai ChuaGilles De HollanderSenta GrafGilles DuverlieSenthilkumar SankararamanGilles WalchSenthilnathan PalaniyandiGillian M. BlueSeockhui KangGimeno MercedesSeok Joo HanGiordano PasqualeSeok Ju SeongGiorgia BeffagnaSeong Hoon BaeGiorgia CaruanaSeong-Kyu KangGiorgio CallerisSeong-Soo ChoiGiorgio CalloviniSeongwoo KimGiorgio HallaertSeon-Yong JeongGiorgio I. RussoSerafín M. MoralesGiorgio LofreseSerena ArrigoGiorgio NovelliSerena CappuccioGiorgio SantoroSerena DuchiGiorgio SonninoSerena ScarpelliGiorgos AlevizopoulosSerena VitaGiovanna BerardiSerenella SerinelliGiovanna CannasSerge ResnikoffGiovanna De MicheleSerge SicouriGiovanna ManziSerge TimsitGiovanna VermiglioSergey KozyrevGiovanni A. RivaSergey TadtayevGiovanni AudinoSergey V KovalchukGiovanni Battista ForleoSerghei CebotariGiovanni BertoldiSergi CesarGiovanni BrandiSergio B. CogliattiGiovanni C ActisSergio De SalvatoreGiovanni CannarozzoSérgio Filipe SousaGiovanni CannavielloSergio FurgiueleGiovanni ChiariniSergio GarciaGiovanni CochettiSergio García-BlasGiovanni ColonnaSergio HaimovichGiovanni ContiSérgio P CamõesGiovanni CorradoSerguei KozlovGiovanni CrisafulliSeth M. TarrantGiovanni D. TebalaSeth NorrholmGiovanni D’AgostinoSetor K. KunutsorGiovanni Dell`Aversana OrabonaSeung Hwan KimGiovanni FalisiSeung Tae LeeGiovanni FilocamoSeung Yong ShinGiovanni GaetaSeung-Baik KangGiovanni GerbinoSeung-han KimGiovanni MarascoSevcan Kurtulmus YilmazGiovanni MauriSeverin RodlerGiovanni MonniSevim AhmedovGiovanni MunzSezgin SahinGiovanni NanoShad B. SmithGiovanni PapaShafiq Ur RahmanGiovanni PerettoShahab GhafghaziGiovanni SansoeShahrul SarbiniGiovanni TarantinoShailendra DasGiovanni TisciaShailendra SinghGiovanni TossettaShailvi GuptaGiovanni TrisolinoShalini ArunogiriGiovanni VitaleShamik ShahGiovanni William OliverioShaminie AthinarayananGiovannino SilvestriShamroop Kumar MallelaGisella VischiniShams Y-HassanGiuditta MannelliShamsah KazaniGiulia BorgheseShamus KeelerGiulia CantiniShane A. BobartGiulia CornoShannan R. TujiosGiulia DegiacomiShany ShermanGiulia Di PrimaShaobin WangGiulia DondiShaoping HouGiulia MidenaShaoqiu HeGiulia OttavianiSharmila FagooneeGiulia SancesarioSharmilee GnanapavanGiulia SchiroliSharna MathieuGiulia TurriSharon AndersonGiuliana FavaraSharon BarakGiuliano CostaSharon CobbGiuliano ZabucchiSharon G. LynchGiulio CoccoSharon K. HunterGiulio FrancoliniSharon ReesGiulio OlivieriSharon ReidGiulio RossiSharon RennerGiulio RussoShatavisha DasguptaGiulio TessarinShaul LevGiulio VernaShawn FarrokhiGiuseppe A. PalumboShazia Mehmood SiddiqueGiuseppe AmorusoShebli AtrashGiuseppe AndòShehzad RehmanGiuseppe ArenaSheila SivamGiuseppe BandiniSheila SpadaGiuseppe BarlettaSheng LiGiuseppe BiaginiSheng ZhuGiuseppe Carlo IorioSheng-Mou HsiaoGiuseppe CasalinoSherouk FoudaGiuseppe CittadiniShibu MathewGiuseppe ComentaleShidou ZhaoGiuseppe D’AngeloShigefumi OkamotoGiuseppe Emmanuele Uma-naShigehiko OgohGiuseppe FaggianShigeki SuzukiGiuseppe Francesco Sferrazza PapaShigemitsu SakumaGiuseppe G. LoscoccoShih-Chang HsuGiuseppe IatíShih-Hsin WangGiuseppe La RoccaShihlung ChenGiuseppe Lo GiudiceShihoko Komine-AizawaGiuseppe MaccagnanoShihori TanabeGiuseppe MarraroShilpa SinghGiuseppe MasciaShilpa ThakurGiuseppe MigliaraShima FukuokaGiuseppe NassoShingo YanoGiuseppe PapaliaShinichi IwasakiGiuseppe PascarellaShinichi OtaGiuseppe Potrick StefaniShinichi SatoGiuseppe PriviteraShinichiro MorichiGiuseppe RinonapoliShinji FukuiGiuseppe RivaShinji KatsuragiGiuseppe SantarpinoShinji TakahashiGiuseppe SignorielloShinji TeramotoGiuseppe StabileShinta NishiokaGiuseppe VezzoliShintaro FujiharaGiuseppe ZimmittiShintaro NakanoGiuseppina AndreottiShinya GotoGiuseppina E. GriecoShinya OkazakiGiuseppina LanzafameShir Lynn LimGiuseppina MalcangiShiro UrayamaGiuseppina OrtuShivam MehtaGiusy DiellaShivshankar ThanigaimaniGlenda Giorgia CaputoShizheng HuangGlenda GobéSho KanataGloria AngelettiShokei MatsumotoGloria FragaShoko KureGloria PelizzoShoumyo MajumdarGniewko WięckiewiczShreyak SharmaGo AnanShruti ChandraGo KanzakiShuang‐Xia ZhaoGodard C. W. De RuiterShubhada K ChotheGokul KrishnaShuichi YamadaGongyi ZhangShuji OzakiGonzalo VarelaShujiro MinamiGoo-Hyun MunShungo ImaiGöran LindShunichiro TakezakiGötz FriedrichShunji OshimaGourav BanerjeeShunsuke KiuchiGözde Şengül AyçiçekShunsuke KurodaGrace A.Llison MastersShunsuke OmotoGrace S. LeeShuta UshioGraeme DukeShuzo SatoGrasiele SausenSi Hyung LeeGrayson W ArmstrongSian Andrea WilliamsGraziano OnderSiddharth SunilkumarGraziela BatistaSiegmund LangGrazyna LietzauSigne Stelling. RisomGregor S. ZimmermannSigrid BraekkanGregory J GolladaySigrun A.J. SchmidtGregory J LandrySihong SongGregory L. BockSilke SchwormGregory NasonSilverio RotondiGregory P. BartonSilvia A. OddoGregory WhitleySilvia BettiniGrete BratbergSilvia CataneseGrigoris EffraimidisSilvia FallariniGrisel J. LopezSilvia FilippiGrzegorz BielecSilvia MagnaniGrzegorz CieslarSilvia Martinez FerreiroGrzegorz GrześkSilvia MascoloGrzegorz M. KozeraSilvia RibbackGrzegorz NowickiSilvia RocchiccioliGrzegorz PiechaSilvia SpotoGrzegorz TrybekSilvio AntoniakGrzegorz ZielińskiSilvio MaringhiniGuadalupe Molina TorresSilvio QuickGuadalupe T. González MateoSimon A. JoostenGuangzhao LiSimon AnnaheimGuenter K. NoéSimon BackhouseGuglielmo Actis DatoSimon BaunwallGuido DohmenSimon DannerGuido PrimianoSimon E. SkalickyGuido SciaudoneSimon John DaviesGuido WernerSimon JS CameronGuido WoesteSimon MastbergenGuido ZavattaSimon NewmanGuilaine BoursierSimon SchoechlinGuilherme Piovezani RamosSimon ThomsonGuillaume BeschSimon WabitschGuillaume BonnetSimona BernardiGuillaume E. CourtoySimona BorghesiGuillaume PortierSimona DEDONIGuillem GruartmonerSimona MartinottiGuillem Muntané-CarolSimona PagliucaGuillermo García-AlíasSimona PergolizziGuillermo PlazaSimona RollaGuinevere GraniteSimona SaccoGuishuang YingSimona SperlonganoGuja AstreaSimona ZaamiGunnar BergenholtzSimon-Dominik HerkenarthGünther EgidiSimone Alex BagagliaGüntülü AKSimone AmbrettiGuram ImnadzeSimone BirocchiGurpreet GandhokeSimone DillonGursukhmandeep Singh SidhuSimone GrafGurupreet SethiSimone MorselliGustavo FernandesSimone NataliGuy FriedrichSimone PerniolaGuy GloverSimone PersampieriGuy Van CampSimone PompeiGuya Diletta MarconiSimone SavastanoGwang Ha KimSimone ScheithauerGwendolyn M. CramerSimone SerafiniGwenhael ColinSimone SforzaH. Christian WeberSimonetta BaraldoHabil Pakai AnnamáriaSimonetta MorselliHadar RosenSimonetta VivianiHae Woong ChoiŠimons ŠvirskisHae-Young LeeSina M. HulkkonenHafeez FaridiSinisa MarkovicHaifa Kathrin Al-AliSiobhan StynesHaitham SaeedSisitha JayasingheHaiyan GongSivabaskari PasupathyHaiyan ZhouSivaraman PrakasamHak ChangSiwei ZhangHak RheeSixto García-MiñaúrHakan KaymakSkaidra ValiukevičienėHåkan N. PärssonSławomir CisieckiHalil İbrahim ÇiftçiSławomir KasperczykHallie ThomasSlawomir TeperHamid MerdjiSławomira Drzymala-CzyzHamza El HadiSmaragdi MarinakiHan Naung TunSmarajit ChakrabortyHana NůskováSneha GautamHanako IkedaSnezana P. PolovinaHanane BouchghoulSobuj MiaHandoo RheeSofia P. RebeloHanna AugustyniakSohan SethHanna Bis-WencelSohee JeonHanna BurkhalterSohee LeeHanna DanielewiczSohsaku YamanouchiHanna M. Koseła-PaterczykSohyun ParkHannah ClaphamSok-Ja JanketHannah StevensSol MarcosHanna-Mari BaldaufSomchai AmornyotinHannu NieminenSonali NashineHans ChristiansenSonali V ThakrarHans Friedrich FuchsSonam KumariHans P. SviggumSong Joo LeeHans StrickerSonia BuschHans TimmermanSonia D'ArrigoHans-Christian DeterSónia Matilde Fonseca Ma-teusHans-Christian SchuppeSonia Ortiz-PeregrinaHans-Gert HeuftSonia SolomonHans-Jonas MeyerSonja KarstHans‐Jörg WittsackSonu Menachem Maimonides BhaskarHanvedes DaovisanSoo Yong LeeHany ElmariahSoo-Hwan ByunHaoSu ZhangSoon Kil KwonHaquima El MourabitSophia LionakiHaralampos KaranikasSophia M. SchmitzHarald De CauwerSophia VassilopoulouHarald KrentelSophie I. MavrogeniHarald SchulzeSophie TaylorHarant HannaSoranobu NinomiyaHarish ChanderSøren Baarsgaard HansenHarish PatelSøren JepsenHarm MaarsinghSøren Ohrt-NissenHarold C. PilsburySøren Paludan SheikhHarold L. RekateSoren U. NielsenHaroon MajeedSorin GiuşcǎHarriet MorfSorin MihaliHarriet ShannonSoshi SamejimaHarry W. SchroederSoumali RoychowdhuryHarshad DevarbhaviSourav PatnaikHarshvardhan SinghSouska ZandiHartmut ImgartSoyoun UmHaruhiko SatoŠpela KonjarHarumi HottaSpilios ManolakopoulosHarvey CoatesSpiros EfthimiopoulosHarvey SarnatSpry ChristinaHarvinder Singh ChhabraSpyridon SiafisHasnaa JalouSravya P. VajapeyHassan KassassirSreedevi AithalHassan TahirSreelakshmi PanginikkodHasse Møller-SørensenSridevi R PittaHatem El-ShantiSrihari KonduriHatice KumruSrinidi MohanHatice TankisiSrinivasa Reddy BonamHauke ThomsenSriramana KanginakudruHayan KwonStaci L. SudengaHaydeh PayamiStamatia TheodoridouHayden L. SmithStamatis GregoriouHayder HashimStamatis KarakonstantisHayley M ReynoldsStanislaw TubekHayley Treloar PadovanoStanley ChanHayoung ChoiStanley W. AshleyHeather M. HowerStavros KalogiannidisHeather StefanskiStavros TryfonHeba AlkhashnamStavros ZanosHector Guadalajara-LabajoStavroula ParastatidouHee Sun KimStefan AcostaHeeyoung LeeStefan BourgeoisHeidi JanssenStefan EversHeike ArgstatterStefan GreinerHeike RockmannStefan HackerHeiko LierStefan HeschlHeiner WoltersStefan J. LangHeinrich GerdingStefan KreuzerHeinrich WeberStefan Kurath-KollerHeinz V. KöhlerStefan MagezHelen HarcombeStefan Marian FrentHelen KoechlinStefan MöhlenkampHelen LileyStefan NaydenovHelen M FeltovichStefan ReichertHelena M. SchotlandStefan RohdeHelena MartynowiczStefan TanglHéléna MosbahStefan WeberHellen NabucoStefania ChiappiniHelmut DeisslerStefania EspaHelmut FriessStefania FerrettiHelmut FrohnhofenStefania MarcuzzoHelmut G. WeißStefania MarsiliHemant Raj MutnejaStefania OttavianiHendrik KohlhofStefania PrincipeHendrik VilstrupStefania RonzoniHengameh Chloe Mirsepasi-LauridsenStefania ZampieriHenk GieleStefanie SamietzHenk Maria KoningStefanie SteigerHenning ReisStefanie-Dorothea KrämerHenrik BjurstenStefano BallestriHenrik C. BäckerStefano CatalanoHenrik FoxStefano CiardulloHenrike ArfstenStefano DamianiHenrique SilvaStefano DastoliHenry Dushan E. AtkinsonStefano De ServiHenry HuangStefano Francesco CrinòHenry PengStefano MalinverniHenry PleassStefano Marco Paolo RossiHenry SutantoStefano MariniHenryk DregerStefano MartinaHerbert MaierStefano MazzaHerbert PichlerStefano MinistriniHerko GrubitzschStefano PaganoHerman Locsin HedrianaStefano Piero Bernardo CioffiHermann WriggeStefano PontoneHermann-georg HolzhütterStefano RamatHermina JakupovićStefano RauseiHernán D. Mejía-RenteríaStefano SacchiHervé FaletStefano TuriHervé ReychlerStefano ZonaHerwig CerwenkaSteffen GrautoffHesam H. MirmohammadiSteffi M. UrbschatHester BanierinkStelios TigasHester VlaardingerbroekSten HellströmHetzel ChristianStephan KrähenbühlHideaki KawabataStephan MarschHideaki OkumichiStephan Marticorena GarciaHidehiro OkuStephan VogtHidehito KimuraStephane CarlierHideki FujiiStéphane MarotHideki KobayashiStephanie A. Eyerly-WebbHidemi NakataStephanie Ducharme-BenardHidemichi KouzuStephanie F. LingHidenori KomiyamaStephanie J. SidowHidenori TakahashiStephanie MarkovinaHideo WadaStéphanie SaxerHidetaka HamasakiStephanie Shoop-WorrallHidetaka NomaStephanie StanfordHideto KamedaStephanie Van BiervlietHideyuki KandaStephen A BernardHideyuki SasakiStephen A. ContagHilary LonghurstStephen B. KayeHilda E. GhadiehStephen CornishHimanshu DeshwalStephen E. ConglyHimanshu GogoiStephen FavaHiroaki IdaStephen GerferHiroaki NakashimaStephen GinsbergHiroaki ShimaStephen I. StoneHiroaki TanakaStephen J RichardsHirofumi HiokiStephen JanzHirofumi ImaiStephen P. BadhamHirofumi ShibataStephen P. WrightHirohito IchiiStephen PolyakHiroki KabataStephen ReingoldHiroki KitakataStephen RobinsonHiroki KokuboStergios BoussiosHiroki NakanoStergios DoumasHiroki TanabeSteve CarlanHiroki TeragawaSteve MilaneseHiroko Kiyoshi-TeoSteve Simpson-YapHiroko KobayashiSteven B. MarstonHiromi MatsubaraSteven D. ColquhounHiromichi YumotoSteven De ReuverHironaga SatakeSteven F. GameiroHironobu TodaSteven F. T. ThijsenHironori IshiguchiSteven J. LavineHiroshi FujishimaSteven M. SullivanHiroshi FukushimaSteven McKenzieHiroshi ImamuraSteven N. LevineHiroshi IrisawaSteven NisticòHiroshi KimuraSteven O'ReillyHiroshi NakaseSteven P. BischHiroshi OkadaSteven P. ShamahHiroshi OueSteven R. FeldmanHiroshi TobitaSteven W. MesHiroshi UreshinoStewart ChristieHiroshi YonekuraStewart R. LakeHiroshi YoshidaStomeo FrancescoHirotaka TanabeStuart JohnsonHiroteru KamimuraStuart LeskeHiroto TsuboiStuart McCluskeyHiroyuki FukuiStuart R. SeiffHiroyuki NiidaStuart RundleHiroyuki TakamaruStuti MisraHiroyuki TorisuStylianos E. OrfanosHiroyuki UeharaStylianos KorasidisHiroyuki WakiguchiStylianos PanopoulosHiroyuki YamadaSu Jin KimHisanaga HorieSu MetcalfeHisashi ShinoharaSubbaramiah SridharHitoshi KobataSubodh K. RegmiHitoshi MinamiguchiSu-Boon YongHiu-yee KwanSubramanian RamakrishnanHiva AlipourSubuhi KaulHo NamkoongSudha SrinivasanHolger F. R JentschSudhakar VenkateshHolger HinrichsenSudi PatelHolly HillSudipta BiswasHong Seok MoonSu-Ho LimHong-Gam LeSujana DontukurthyHoon Joo YangSujata MohantyHorea FeierSu-Jung ShinHoria BumbeaSukhmani BediHorst OlschewskiSu-Kiat ChuaHosein Hoseini-YazdiSuk-Tak (Phoebe) ChanHossam AliSukvinder K. BhamraHossein TaghizadehSumadi Lukman AnwarHou-Chuan LaiSumali PandeyHoussam FarresSuman DuttaHoward LevinsonSumana ChintalapudiHsien-Yuan LaneSumana GhoshHsin-An ChangSun Hwa LeeHsin-Yu HoSun ImHsu-Heng YenSun Mi ChoiHuahao FanSun Woong KimHubertus AxerSun Young LeeHugo R. MartinezSunan LiHugues GeorgesSundar RamalingamHui WangSundararajan JayaramanHui ZengSuneel KumarHuize PanSung Eun KimHülya KarataşSung Mi HwangHun LeeSung Sam GongHungwen LinSung Yong OhHungwon TchahSunghoon ParkHüseyin InceSungjin ChungHussein AbdelazizSung-Kyo KimHye SeongSungmin LimHyechang RhimSungmok JungHyeong Won YuSung-Woon OnHyo-Joon YangSungyeon HamHyo-jung JeongSunil KarhadkarHyoun KimSunil KimHyuck Min KwonSunil KurianHyuk-Joon LeeSunita KeshariHyuk-Joong ChoiSun-Joo JangHyun Jin ChoiSupreet AgarwalHyung Joon SeoSurasak SangkhathatHyung-In YoonSuresh SamsonHyungjong ParkSusan ChristoHyungju KwonSusan L. HutchinsonHyungkeun KimSusana Al-HalabíHyungon LeeSusana FerreiraHyun-Mo RyooSusana Núñez-PereiraHyun-sun ParkSusanna Cunning-ham-RundlesHyun-yoon KoSusanna KullbergIacopo GesmundoSusanna ZuurbierIain McNamaraSusannah MayhewIakovos AmygdalosSusanne KimeswengerIan C FrancisSusumu SatoIan GanlySuwasin UdomkarnjananunIan James MartinsSuzanne ArendsIan L. ValerioSven Arke LangIan MacDonaldSven FrancquevIbolya FülöpSven HoppeIbrahim NassourSven LichtenbergIbrahim OuerguiSven P. HeinrichI-Cheng LuSvetlana BlitshteynIciar ArteagoitiaSwarup MitraIcro MaremmaniSwati JainIda AringerSwayam Prakash SrivastavaIda NilssonSwetha KanduriIdan GorenSybil CharrièreIdoia LarretxiSyed Ali ImranIgnacio Díez LópezSyed MehdiIgnatius CondroSyed Saad B QasimIgor A. ZolotukhinSyed Sayeed AhmadIgor DumicSyed Tabish R. ZaidiIgor GiarrettaSylvain CatrosIgor P. KaidashevSylvain LamureIgor VendraminSylvain SteinmetzI-Jong WangSylvia DrazilovaIker MadinabeitiaSylvia E. PerezIkjoon MoonSylvia FaictIlaria AttiliSylviane DefresIlaria CampioniSylvie Di FilippoIlaria CosciSylvie EpelboinIlaria GandolfiniSylwia MałgorzewiczIlaria GuerrieroSzczepan PiszczatowskiIlaria MullerSzymon BudrejkoIlaria RoatoSzymon SkoczeńIlaria SimonelliT. Charles CasperIlia StamblerTabitha Turner-StokesIlias MigdalisTabito KinoIlias P. DoulamisTa-Chuan YehIlias SmiliosTadafumi AsaokaIm Hee ShinTadakazu KondoImelda Ontoria-OviedoTadamichi AkagiImran T KhawajaTadashi YoshidaIn ChoTadej PetreskiIna Michel-BehnkeTadeja KuretIna NordmanTadeusz IssatIna RudloffTadeusz OsadnikInas F. AboobakarTae Gyun KwonIndu G. RajapakshaTae Ho HongInés Aguinaga-OntosoTae Kyung HaInês FerreiraTae-Gyu LimInês FranciscoTae-Jong KimInês SilvaTahlita C. M. ZuiverloonInger Lise GadeTai Soon YongIngo VolkmannTaichiro GotoIngrid PoulsenTaihei ItoInka MiñambresTaiji NagaokaInken BrockowTai-Li ChenInki MoonTaishi YonetsuInmaculada Martinez-SaguerTaishirou ChikamoriInoue AkiraTakaaki InoueIn-Seop LeeTaka-Aki MatsuyamaIn-Sook KwunTakafumi HosokawaIoan LascarTakafumi KadonoIoan SarbuTakafumi OkaIoan SporeaTakahiro NakajimaIoana Diana OlaruTakahiro NemotoIoanna K. BoliaTakahiro SodaIoanna MylonaTakahisa SuzukiIoannis A. KalogiannidisTakamasa KinoshitaIoannis AnagnostopoulosTakamasa UenoIoannis BoletisTakao ImaiIoannis BoutasTakao SaitoIoannis FarmakisTakashi HaranoIoannis GeorgiouTakashi KamioIoannis HalkiadakisTakashi KanbayashiIoannis KoliarakisTakashi KenmochiIoannis KosmasTakashi KondoIoannis KoutelidakisTakashi KuritaIoannis LiampasTakashi MatsushimaIoannis TsakiridisTakashi YoshiyamaIoannis VourosTakawira C. MarufuIolanda LázaroTakaya HigakiIonut DonoiuTakayuki AnazawaIrena Duś-IlnickaTakayuki IshiharaIrene AragaoTakayuki KatoIrene CacciolaTakayuki SaitohIrene CampiTakehiro IwataIrene E. SchauerTakeji SaitohIrene HarrisTakemi OtsukiIrene RamosTakemichi FukasawaIrene ScaleraTakenori HarunaIrene ScavelloTakeo IsozakiIrene SchiavettiTakeshi AokiIrene SzijanTakeshi KamiyaIreny Y.K. IskandarTakeshi KannoIride Francesca CeresaTakeshi KezukaIrimia Mollinedo-CardaldaTakeshi KikutaniIrina MurakhovskayaTakeshi MurataIrina NesmelovaTakeshi NamekawaIrini P. ChatziralliTakeshi SasakiIris ManorTakeshi UnokiIris Z. UrasTakeshi UradeIrving E. SalitTakeshi YoneshiroIryna SamarskaTaketoshi MizutaniIsaac RuizTakeyuki TanakaIsabel FortTaku SugiyamaIsabel Garcia-GarciaTaku WakabayashiIsabel Mourão-CarvalhalTakuhei ShojiIsabel Muñoz-PousaTakuji TanakaIsabel RodriguesTakuma InagawaIsabel SevillaTakuto HikichiIsabel SilveiraTakuya MaedaIsabelle Galy-FaurouxTakuya NakaiIsabelle MosnierTal Ben-AmiIsaku OkamotoTalha NiazIsam BsisuTamaki IkuseIsao HidakaTamam BakchoulIsao OtsukaTamami OdaiIsmael SeáñezTamara UrsiniIsmaheel O. LawalTamás BaranyaiIsmail SyedTamás MajorIsmini E PapageorgiouTamás VéghIsrael Casado-HernándezTamra RanasingheIssac SachmechiTania López HernándezIssam KoleilatTanja DrewsIstván KertészTanja FalterItalo BraghettoTanja RestinItamar YehudaTanja Schmitz-HübschIto TakahideTanushree DangiItziar EseberriTanveer AhmadIuliana CosteaTanzil Mahmud ArefinIuliana CotiTao ZhangIuliana NenuTara DonkerIuri CorsiniTarek ElshebinyIva SpeckTarek Mohamed Abd El-AzizIvan CundrleTarik Z. AlhmoudIvan De MartinoTariq AzamIvan GiovanniniTarja TannerIvan KopitovićTaro HorinoIvana BurićTaro MatsukiIvana GiangriecoTarr TündeIvana SamarzijaTateaki NaitoIvo BeverinaTatenda C. NzenzaIvo GuberTatiana SidiropoulouIvo M Van DongenTatijana ZemunikIwona GorczycaTatjana SimićIwona ObuchowskaTatsuhiro MasaokaIzabela DomitrzTatsuhiro SatoIzabela ZakrockaTatsuo KandaIzabella Karska‐BastaTatsuo ShimosawaIzabella PawluczykTatsuo ShirotaIzabella UchmanowiczTatsushi MutohIzumi KajiTatsuya AbeIzumi NakayamaTatsuya NakamaJ. Christoph KatthagenTawna L. RobertsJ. Clay BavingerTaylor HatchardJ. Daniel TwelkerTea GalicJ. De MouzonTeake P. EttemaJ. Javier Gómez-RománTeayoun KimJ. Marc SimardTeddy CaderbyJ. Pedro TeixeiraTeijiro HirashitaJ. S. Hill GastonTejal AminJaana NevalainenTeodora DonisanJacalyn McCombTepetes KonstantinosJacek BoguckiTeppei SasaharaJacek GrzybowskiTeppei TakeshimaJacek KubicaTerence OngJacek NasiłowskiTerence PayneJacek PiątekTeresa GavaruzziJacek SmerekaTeresa PaolucciJacek SzymańskiTeresa PerraJack AnsellTeresa SalvatoreJack StapletonTeresa SommaJackie Tan-ShowyinTerry SwansonJaclyn StephensTerry-Elinor ReidJacob BarTeruhiko ImamuraJacob E. OllechTeruyo KidaJacob F CollenTessa CoppJacob FreedmanTessho MaruyamaJacob GeniziTetsuhiro YoshinamiJacob MashiahTetsuji AoyagiJacobus MullerTetsuro IkebeJacobus PrellerTetsuya AmanoJacobus Van Der VeldenTetsuya HirataJacopo AgrimiTetsuya SugiyamaJacopo CiaffiTetyana YevsaJacopo GiulianiThais Pereira-VeigaJacopo Maria LegramanteTheirry BerneyJacques AbramowiczThelma De Aguiar PertinhezJacques HimpensThemistoklis MikosJadranka Buturović PonikvarThemistoklis TsatalasJae Gyu KimTheodora PapamitsouJae Ho LeeTheodore EliadesJae Hui KimTheodore H. WeinJae Min ChoTheodore KarapanayiotidesJae Sung LeeTheodore TowseJae Wook YangTheodoros FilippopoulosJae Yong KimTheodoros I. RoumeliotisJae Young ChoiTheofani AntoniouJaechan LeemTheophile GuilbaudJaehong AhnTheresa Harvey-DunstanJae-Hyeong ParkTheresa V. StrongJae-Jin KimThereza Maria Magalhães MoreiraJae-Joon HwangThiago Saads CarvalhoJae-Kwang ShimThibaud GarcinJaesung ChoThibault TricardJae-Woo ChoThiebaud PicartJaeyoung KimThiên-Nga Chamaraux-TranJae-Young KimThierry LeblancJagannadha R. AvasaralaThierry PrazuckJahnavi AluriThilo WelschJai Young ChoThinzar MinJaime Del RíoThomas B. PoolJaime EscallonThomas BartlJaime EspinozaThomas BaskinJaime Piquero-CasalsThomas CluzeauJakob Hartvig ThomsenThomas FrauenfelderJakob JosiassenThomas FrielingJakob K. AnningaThomas HarrerJakob Starup-LindeThomas J. BaumgartenJakob WollbornThomas J. GastJakub GumprechtThomas J. MartinJakub HadzikThomas KoudstaalJakub KaźmierskiThomas KühneJakub KortasThomas LafonJames AlibhaiThomas LambertJames A.M. ShawThomas LiebigJames Cheng-Chung WeiThomas M. HofbauerJames E. NeffendorfThomas M. SuszynskiJames HongThomas M. TiefenboeckJames HurleyThomas MairingerJames IvoryThomas MalinkaJames K. RussellThomas MonaghanJames KarantonisThomas MudersJames KimballThomas MüellerJames Kit-hon TsoiThomas NeumannJames M. SandersThomas NührenbergJames McFadyenThomas OchsenkühnJames McNamaraThomas P. DavisJames PethickThomas Patrick LillicrapJames PowersThomas PerreaultJames R. BrorsonThomas RamppJames R. GossageThomas RückschloßJames R. S. HockerThomas SenonerJames RowleyThomas SonnweberJames V. AquavellaThomas StaudingerJamie Kaye BurgessThomas StiermaierJamie PenkThomas ThomsenJan Adriaan GrawThomas Van GulickJan BewersdorfThomas Von HahnJan B.F. HulscherThomas ZegkosJan BoublikThomasz M. GondekJan BullaThor BechsgaardJan Duedal RölfingThorbjorn S. R. JensenJan HartvigsenThorsten BraunJan HenzelThorsten DiemerJan HoflandThorsten RudroffJan Jens KoltermannThucNhi Tran DangJan KassubekTiago DiasJan KotarskiTiago GregórioJan KrogTianhao (Vincent) ZhouJan Krzysztof NowakTianji ChenJan LexellTianjun WangJan Paul MulierTianyi HuangJan PetriczkoTiffany PattersonJán RemšíkTigestu Alemu DesseJan SvihraTihamer MolnarJan TesarikTihomir Georgiev-HristovJan Van SchaikTiina KelkkaJan Walter SchroederTill DammaschkeJan WesterinkTilman CalliessJan Willem KallewaardTilman EmrichJan ZabrzynskiTilman ZieglerJana KatuchovaTim R. GlowkaJana NekolováTim RosenowJana SchmidtTim StuckenschneiderJane A. LeopoldTim WalkerJane OliverTim WehnerJane SimonsenTimmy LiJaneen R. JordanTimo EppigJanet JamiesonTimo F.W. SoeterikJanet Snell-BergeonTimo GaberJanewit WongboonsinTimo MeineJaney ProdoehlTimo RathJanice Jin HwangTimothy C. FaganJanin HenkelTimothy E. O'BrienJanina StepinskaTimothy N. ClintonJanine LenkTimothy PruettJanis Marc NoldeTimothy R. KeifferJanka BábíčkováTimur SellmannJanna G. KoppeTina Dam KristensenJanni Vikkelsø JeppesenTina In-AlbonJannos SiaplaourasTina MckayJanos NemethTin-chiu LiJanos Tibor KisTine D. ClausenJanusz Konstanty-KalandykTine Plato HansenJanWillem DuitmanTino MünsterJared J. BarrottTirayut VilaivanJarmo OksiTissa WijeratneJaroslav VadlejchTiziana CavalliJarosław BilińskiTiziana PietrangeloJarosław ĆwikłaTobias BecherJaroslaw RegulaTobias BerglerJasmina ProfirovicTobias F.S. PustjensJasmine ElisonTobias KalischJason M SouzaTobias M. BallhauseJason S. ChinitzTobias PiegelerJason S. RadowskyTobias RufJason TasoulasTobias SchuppJason Yongsheng ChanTobias SchwarzJaume Alijotas-ReigTobias StrunzJaume VivesTobias WinklerJavier AlonsoTobias ZimmermannJavier C. AnguloToby EyreJavier Garcia-AlbaTom CoxJavier H. CamposTom SchepensJavier LaverniaTomas BlancoJavier Martinez-SanzTomás José González-LópezJavier Molina-CerrilloTomas MustelinJavier RosTomas SimurdaJavier SantabarbaraTomáš ToporcerJavier Sanz-ReigTomaso BottioJay KarriTomasz BednarczukJay N. PatelTomasz BondaJay WangTomasz CudejkoJaya ShrivastavaTomasz DziodzioJayanthi ParthasarathyTomasz FrączykJayson R. BamanTomasz GołębiowskiJayson StoffmanTomasz HawroJe Hyeok OhTomasz JadczykJean AmiralTomasz KotwickiJean BertoldoTomasz LitwinJean Claude AldigierTomasz M. KarpińskiJean DemarquoyTomasz MarjanskiJean Gabriel CharchafliehTomasz PłoszajJean Louis VincentTomasz PorażkoJean Luc PaganiTomasz RakowskiJean Marc PeroneTomasz RusakJean Marie GraïcTomasz SawczynJean P. Li-Kim-MoyTomasz SzmudaJean SenemaudTomasz TokarekJean ThérouxTomasz UrbanekJean-Baptiste DucloyerTomasz ZarnowskiJean-Baptiste PinaquyTomasz ZielińskiJean-Baptiste RiccoTommaso BacciJean-benoit ArletTommaso BucciJean-Christophe OrbanTommaso FasanoJean-Damien RicardTommaso FossaliJean-Daniel LalauTommaso PellisJeanette C. MostertTommaso PettenuzzoJean-François BernaudinTommaso RossiJean-François LesesveTommaso VerdinaJean-Francois ReyTommy BaumannJeanine R. Jarnes-UtzTomoaki AndoJeanine RoodhartTomoaki YohJean-Louis BayartTomofumi NishinoJean-Louis PujolTomohiko OzakiJean-Marc SellalTomohiro OishiJean-Marie ExbrayatTomokazu ShimizuJean-Michel PawlotskyTomoki NakamizoJeanne Du Fay De LavallazTomoki NakamuraJeanne M. Du-Fay-De-LavallazTomoki ShimokawaJeannette Ossewaarde-van NorelTomoko HayashiJean-Philippe DidonTomoko MatsumotoJee Hye WeeTomonobu AbeJee-Young MoonTomonori KenmokuJeffrey A. BerinsteinTomoo KishabaJeffrey A SoRelleTomoya OkazakiJeffrey DalliTomoya TateishiJeffrey DavidsonTomoyasu KadoguchiJeffrey J. HuangTomoyuki KabutoyaJeffrey LuttrullTomoyuki KawamuraJeffrey M. OsgoodTomoyuki KunishimaJeffrey S. ForsseTomoyuki MukaiJelena DjordjevicTonino BombardiniJelena PrpicTono TetsuyaJennifer A. DavidsonTony AbdoJennifer A McCaughanToon DuijnhouwerJennifer Ann LockeTorbjörn LedinJennifer C. BoerTore AmundsenJennifer F. GerardinTorto Federico. LoJennifer FangToru IshiharaJennifer GlausToru NabikaJennifer Joan RyanToru YagoJennifer Lagoutte-RenosiTorunn Hatlen NøstJennifer M. DewingToshiaki KubotaJennifer PhyToshiaki SaidaJennifer R. Stapleton-KotloskiToshifumi KinJennifer SalinasToshihiko TashimaJenn-Ming YangToshihiro KitaJenny ChenToshihiro MurajiJenny DoustToshihiro TsurudaJenny SherriffToshikazu KimuraJens P. PanseToshiki MiuraJens Rolighed LarsenToshimasa SoneJeonghoon HaToshinobu KazuiJeong-Hun ShinToshio MatsumotoJeongkui KuToshitaka YamakawaJeong-Yeol ParkToshiya NishikuboJeppe LangeToshiyuki KawaiJeremias BayonToshiyuki MatsuiJeremie D. OliverToshiyuki SetoJeremy JohnsonTossaporn SeeherunvongJeremy LagrangeTova RonisJeremy Robert ChapmanTove WästerlidJeroen J.H. BungeTracy FrechJeroen Van Der HeijdenTriana LobatonJérôme AddaTriantafyllos DidangelosJérôme CosteTristan Morichau-BeauchantJerome Gay-QuéheillardTrung T. NguyenJerome LowensteinTsuchiya TakayoshiJérôme RollinTsukasa IshidaJerónimo GonzálezTsuneaki KenzakaJerzy MosiewiczTsung Chieh Chester LinJerzy NarlochTsutomu NishidaJerzy ŚwierkotTsuyoshi HamadaJerzy WiciakTsuyoshi IchinoseJess LambrechtsenTsuyoshi ShimamuraJesse GroenTsuyoshi YamaguchiJesse R. QualliotineTu Anh DuongJesse RomanTuck YongJesse VeenstraTuija MännistöJesse Yoe Rumbo MoralesTung Yu TsuiJessica A. WilliamsTuomas O. KiviniemiJessica BarlinnTurgut B. CengizJessica HardingTzu-Yu PengJessica HuaUberto BortolottiJessica J. TuanUdi VazanaJessica R. SimonUgo ConsoliJessica RademacherUgo CovaniJessica S. WhittleUgo FalagarioJesús Mena-ÁlvarezUgo GrossiJesus PelaezUgo IndraccoloJesus PeteiroUgolino LiviJeungchan LeeUlas̈ CıklaJezabel BlancoUlf GuentherJ.H.C. (Joost) HeutinkUli FehrenbachJia WuUlla Breth KnudsenJia YuemengUlla KampmannJia-Feng ChangUlrich Fischer-RasokatJianfeng LiUlrich SachsJianjun WenUlrich WalterJianli HuUlrika SandvikJian-Min ChenUmang SwamiJiao WangUmar HayatJiaxing WangUmbert BarberoJie GaoUmberto AnceschiJiesuck ParkUmberto BaccaraniJigar ModiUmberto BasileJignesh K. PatelUmberto CucchiniJill WaalenUmberto TarantinoJillian BrownUmer DaoodJillian ClarkeUn Chul ParkJillian H BroadbearUnai Perez-SautuJillian R. RichterUnzué LeireJimmy SundblomUri AlkanJin Ha ParkUrvish PatelJin JooUsama ElewaJin WeiUte SeelandJin Young NamUtkarsh KohliJin ZhangUwe Von FritschenJinbo HeV Carroll PaulJing J. WangV. Ram PeddiJing LiVadim KlimontovJing SongVadim VasilyevJing ZhangVagner Oliveira Carvalho RigaudJin-Hwa JungVahan KépénékianJinkwon KimVaithinathan SelvarajuJin-Seok KimValay A. ShahJiří HanáčekValder ArrudaJiri PlasekValentin HerberJiro KishimotoValentina Brzović RajicJithendra T. B. RatnayakeValentina BucciarelliJiwei GuValentina CaputiJiwon M. LeeValentina Di DonnaJiwon RyuValentina GiudiceJi-Yeob ChoiValentina GrazioliJiyeon KangValentina LanteriJi-Yoon NohValentina LongoJo Andre Y. DensValentina ParmaJo KatoValentina PinziJo M. ZelisValentina RovelliJo NijsValentina SalaJoachim Friedrich KueblerValentina VicennatiJoachim JähneValentino ConterJoachim KlischValentyn StadnytskyiJoachim LupbergerValer DzupaJoan Albert BarberàValeria BrazzelliJoan BagóValeria CentoJoan CidValeria CorradettiJoan HowValeria ManganelliJoan Leal-BlanquetValeria MerzJoan Marti-FabregasValeria PergolaJoan Torras-AmbròsValeria SimoneJoana CamposValerian DormoyJoana MesquitaValerie B. WeihsJoanna A MusialikValerie ChappeJoanna Bartkowi-ak-WieczorekValérie SautouJoanna BladowskaValerio D'OraziJoanna BogusławskaValerio MaisJoanna J. WykrzykowskaValery V. LikhvantsevJoanna KonopińskaValter LubranoJoanna M. WardlawValtteri UusitaloJoanna MazurVance Girard NielsenJoanna PawlakVanessa BianconiJoanna Pet-ryka-MazurkiewiczVanessa BrébantJoanna ReszecVanessa SmithJoanna RogVasileios PapavasileiouJoanna RógVasilije StambolijaJoanna Rupa-MatysekVasiliki SiozopoulouJoanna SherwoodVasiliki YotsidiJoanna SiudaVassilios PapaloisJoanna StefanowiczVassilis L. TzounakasJoanna StragierowiczV.E. OleynikovJoanna Wasielica-PoslednikVegard Asgeir ForsaaJoanna WróblewskaVegard Heimly BrunJoanna ZawitkowskaVenera TomaselliJoanne MurrayVenerando RapisardaJoanne R. MorlingVenkatakrishna Tholaka-nahalliJoanne Van RynVenkateswara Reddy GogulamudiJoão André FerreiraVera ColomboJoao Barbosa-BredaVera SchwierzeckJoao Ferreira-CoimbraVered H. EisenbergJoão MartinsVerena ScheperJoão Nobre CardosoVerena SchönauJoão Ramalho-SantosVerena WallyJoão RodriguesVeronica AbateJoao Santos-AntunesVeronica Alexandra AntipovaJoaquin GadeaVerónica Gómez-GilJoaquín López-ContrerasVeronica Perez-CabezasJodi HedgesVeronica SansoniJoe Truong NguyenVeronika AleksandrovychJoël Fokom DomgueVeronika Buxhofer-AuschJoel GelmanVeronika VielsmeierJoel JamesVéronique Le Cam DuchezJoel NeugartenVesna KosecJoel V. ChuaVicenc Torrente-SegarraJoelle Nsimire ChabwineVicent Primo RomagueraJoerg AlbertVicente Calixto Za-non-MorenoJoerg KrebsVicente ClimentJoffrey PelletierVictor Arufe GiraldezJohan H. C. ReiberVictor CasulaJohan HoebekeVictor Doménech GarcíaJohan NobleVictor GarciaJohann HreinssonVíctor Gómez‐MayordomoJohann SellnerVictor J. Costela-RuizJohanna C. HerkertVictor Jilbert VerwaalJohanna T. KurzhagenVictor LingJohannes BrachmannVictor M. SchuettfortJohannes E. PlathVictor M. VictorJohannes F. PlateVictor SendraJohannes FrankVictoria G. RontoyanniJohannes FunkenVictoria MacBeanJohannes GleichVictoria TeamJohannes HoferVictor-Vlad CostanJohannes Hugo SchouwinkVid MatišićJohannes Jakobsen SidelmannVida AbediJohannes LeiererVida DemarinJohannes MagerVidhi ChandraJohannes MairVidula VachharajaniJohannes MuellerViet LeJohannes OppermannViet TranJohannes OttVignesh T. PackiamJohannes SchwabVijay R. VarmaJohannes StadlmannVijay Sagar MadamsettyJohannes UhligVijayakumar SukumaranJohannes ZenkVikash AgrawalJohn A. D’EliaVikrant RaiJohn C. MiddlebrooksViktor DomisloviĆJohn D. GordonViktor HamreforsJohn F. CarlquistVille LeinonenJohn HoughVille PimenoffJohn J. CareyVimal ChadhaJohn JarrellVimala BharadwajJohn K. McGuireVincent CrennJohn K. YueVincent DescampsJohn Kenneth PeelVincent J. AlentadoJohn L. FraterVincent L. ChenJohn L. PhillipsVincent L. RoweJohn LeddyVincent MaidaJohn M. CarneyVincent PonsJohn M. DiBiancoVincent ZimmerJohn MayberryVincenzo De CeglieJohn MercerVincenzo De FrancescoJohn Patrik BurkhardVincenzo Di LeoJohn R. RinkerVincenzo Di NunnoJohn Richard LeeVincenzo Di StefanoJohn W. GittingerVincenzo MarottaJohn William SleasmanVincenzo MicaleJohnny NijmVincenzo MollaceJoJo WongVincenzo SaliniJolanta ArtymVincenzo SavarinoJolanta JaworekVincenzo SorrentinoJolanta Justina VaškelytėVincenzo VigoritaJolanta Klukowska-RötzlerVinicius Tieppo FrancioJolanta Słowikowska-HilczerVinita ChittoorJolanta-Justina VaskelyteViola KnopJon B. SuzukiViola Maria PopovJonas KneerVirginia AlbiñanaJonatan MirandaVirginia CorazziJonathan BarrattVirginia Di PaoloJonathan BenzaquenVirginia LiberiniJonathan CantorVirginie BetinJonathan D. SchofieldVirginie PrendkiJonathan EisengartVisanee TantiseviJonathan EskanderVishal JaikaransinghJonathan GarnierVishnu Muthuraj Ku-marasamyJonathan HewittVishnuprabu Durairaj Pan-dianJonathan HyettVishwaraj SontakeJonathan LinVít WeinbergerJonathan Michael FlanaganVita DolžanJonathan PanVito FioreJonathan RoydsVítor CavadasJonathan S. CalvertVittoria MurroJonathan TroostVittorio FuscoJonathon S. GaniVittorio PiraniJondavid MenteerVittorio RamellaJone TrovikVivian LeeJon-Émile S. KennyVladimir HeiskanenJong Woong ParkVladimir LazarevićJongerius IlseVladimir LeksaJong-Jer LeeVladimir StevanovicJong-Keun SeonVladislav A. VukomanovićJong-Ki HuhVlasta FesslovaJonica CampoloVo Phuoc TuanJoon Hyop LeeVolker KiefelJoon Won KangVolker VielhauerJoop JonckheerW. Paul Farquhar-SmithJoost R. van der VorstWadi N. SukiJordan B. StromWael AbuzeidJordan PatikWakiro SatoJordan S. KlebanoffWalter MihatschJordi Martorell-MarugánWalter NeuJordi RelloWalter WahliJörg MeyleWang Zheng-XinJorge A. Roman-BlasWankun XieJorge AparicioWarwick Jonathan TeagueJorge Cortés-Bretón Brink-mannWataru KikushimaJorge JovenWathik AlsalimJorge Manuel Martins JorgeWayne L. ChandlerJorge Moreno-FernandezWei Shiang LinJorge Moya HiguerasWeicheng ChenJorge N.R. MartinsWei-Chieh LeeJorge PaulinoWellington M. CabreraJorinde Aw PoldermanWenbo ZhangJoris G. De ManWen-Chung LiuJoris Van Der HeijdenWendy Scholten-PeetersJörn A. LohmeyerWenting DuJorrit Jan VerlaanWen-Yee LeeJose A. GuirolaWenying LuJosé Afonso NevesWenzel SchöningJosé Albeto Laredo-AguileraWerner CordierJose Alegre-MartínWeronika GonciarzJosé Ángel Díaz-PérezWeronika StupalkowskaJose Antonio Gonza-lez-FajardoWesley HayesJose Antonio Lopez-EscamezWesley KerrJosé Antonio PintoWhitney BrownJosé António Vasconcelos OliveiraWiktor PaskalJose Di DiegoWilbert AronowJosè Divino-FilhoWillem A. DikJosé Enrique Alés-MartínezWillem LemsJose G. MantillaWilliam B. ClarkJosé Javier Miguel-HidalgoWilliam E. WhiteheadJosé Javier Zamorano-LeonWilliam G. BlakeneyJosé Jesús BrosetaWilliam HurfordJosé Luis Cebríán-CarreteroWilliam Ji Shian HuangJosé Luis Del-Castillo-Pardo-de-VeraWilliam L. IrvingJosé Luis Hernández-VerdejoWilliam LedgerJose Luis Pérez-CastrillónWilliam MorelloJosé Luís Sánchez-CarazoWilliam N. HaJosé M. JiménezWilliam P. McKayJosé M. PortolésWilliam PapaioannouJosé María De Miguel-YanesWilliam R. ReedJose Maria Gonzalez RuizWilliam Sean DavidsonJose Maria Pereira De GodoyWilliam T Gunning IIIJosé Martínez-HernándezWilliam XuJosé Norberto Sancho-ChustWilly HauzerJosé Pedro Lopes NunesWilson I. GonsalvesJosé Peña-AmaroWim CouckeJose Renato PintoWinfried AmoakuJosé TuñónWinifred MakJosé Ursic-BedoyaWinnie ChanJosef SmolleWioletta Szczurek-WasilewiczJosef Stolberg-StolbergWisit KaewputJoséJ Juan IllarramendiWitold GertychJosé-Juan Pereyra-RodriguezWitold OwczarekJoselyn N. AllenWitold TłustochowiczJose-Manuel Ramos-RinconWiwat ChancharoenthanaJosé-María Sánchez-GonzálezWladimir MauhinJosep GallifaW.M. Van Den BerghJosep Maria Fernandez NovellWojciech EliaszJoseph BorrelliWojciech HankeJoseph CarlsonWojciech JachećJoseph L. EdmondsWojciech Jan SkorupskiJoseph LeeWojciech JelskiJoseph P. CraveroWojciech KosiakJoseph P TerlizziWojciech KrajewskiJoseph PlasekWojciech WitkiewiczJoseph SeitlingerWojciech WitkowskiJoseph SiaWojciech WojakowskiJoseph (Sifis) PapamatheakisWolfgang ArztJoseph TestaWolfgang DichtlJoseph W. GalvinWolfgang F. BuhreJosephine Berger-GrochWolfgang FuhlJoshua K. NaporaWolfgang KuepkerJoshua LightfootWon Chul ChoiJoshua M. KolzWoo Hyun PaikJoshua R. SmithWoo-Baek ChungJoshua Y.C. YangWoo-Hyun LimJosy AugustineWooil KwonJózef MierzwińskiWoojin KimJuan Aguilar-CompanyWoo-Keun KwonJuan Antonio Valera-CaleroWoon Yong KwonJuan Carlos Prados FrutosWouter H Van GeffenJuan Delgado-FernándezWybraniec MaciejJuan F. Blanco‪X. Michelle AndroulakisJuan Flores-MonteroXavi Suárez-CalvetJuan FranciscoXavier CorbellaJuan Francisco Sánchez-SolerXavier DurandoJuan Garcías-LadariaXavier Fulladosa OliverasJuan Jesús García-IglesiasXavier GuillonneauJuan José Alegre SanchoXavier MontanéJuan José López-NúñezXavier Navarro-AcebesJuan José Palacios GutiérrezXavier RoblinJuan José Ramos-RodríguezXavier StruillouJuan Lacalzada-AlmeidaXavier TroussardJuan Luis AlcázarXénia LatypovaJuan M. Fernández-CostaXiangjun ZhangJuan Manuel Espi-nosa-SanchezXianqi LiJuan Manuel Marquez-RomeroXiaodong GeJuan Manuel PericasXiaofeng Allen SuJuan Miguel Martínez Gali-anoXiaojia GuoJuan Pedro-BotetXiaotang MaJuan SanchisXiaoyan SongJuan SeoaneXin CuiJuan TorrasXinbo LiJuan Torres-MachoXingjun FanJuan-Pablo IdrovoXinli LiJudith Louise FlanaganXinxing ZhangJudith NissenXiujuan ZhangJudith TateXosé Luis Pérez-FernándezJudith Van Der WaerdenXuejing ChenJudson Vincent EdwardsXuqiao ChenJudyta JuranekYa Fatou Njie-MbyeJuerg KesselringYacov ReismanJuergen GrauvogelYael Shapira-GalitzJuergen RockstrohYael ZaltzJuha TaavelaYahya ShehabiJuhani AiraksinenYahya SohrabiJuhani H. MäättäYaichiro OkuzuJukka P. KoffertYair LotanJules MesnierYan HuangJulia E. Marquez-ArricoYanbo YuJulia FerrariYandong LaiJulia FussellYang GeesuJúlia María Sánchez-SchmidtYang Gyun KimJulia SongYangpeiwei HuangJulián AragonésYanhui HanJulian L. GendreauYanmei HuJulian TaylorYanna CaoJuliana MarottiYanshuo HanJulianne E. ZweifelYao LiJulie Brogaard LarsenYao-Chuen LiJulie C KohnYaohua YangJulie FoxYari LongobuccoJulie Horgen EvensenYasser AlakhdarJulie LeeYasuhiko HayashiJulie RizzoYasuhiko KogaJulie SheldonYasuhiro IchiboriJulie Steen PedersenYasuhiro KuroiJulie VrankenYasuhiro MikiJulien AniortYasuhiro TakahashiJulien DemiselleYasuhito TanakaJulien FromonotYasuhito TeruiJulien GuiotYasuki HoriJulien MarletYasuko FujitaJulien Saint-PolYasumasa YamadaJulio C Zuniga-MoyaYasuo MiyagiJulio Gómez SorianoYasushi ShinoharaJulio Marti-AlmorYasutoshi KidoJumpei SaitoYasutoshi KimuraJun Hwan ChoYasutsugu ShionoJun MikiYasuyuki NakadaJun NakamuraYasuyuki TokinagaJun Neng RoanYawei LiJun OtoYdo Vincent KleinlugtenbeltJunaid AhmedYen Chin KoayJune-Sung KimYeun-Yoon KimJung Hyun KwonYezaz GhouriJung Kee MinYi ShengJung-hee LeeYi SunJung-Hoon ParkYi XiaoJungHwan OhYi YanJunghyun ChoiYi-Chou HouJungwon MinYichun TsaiJun-ichi KawadaYigal HelvizJun-Jun YehYihao ChenJunxuan MaYi-Hsing ChenJunya AokiYi-Jen HsuehJunya FujinoYijun PanJunya KusumotoYiling J. ChengJunya SatoYinchen SongJunya ToguchidaYingli GuJunyan LiYingpeng LiuJunyan TaoYingying TangJunying LiYitang SunJuqian ZhangYi-Ting LinJuraj MokrýYlenia BartolacelliJurij HanzelYlenia DucaJussi KosolaYoav ArnsonJuste KaciulyteYoav KohnJustin BeilbyYoav MazorJustin NguyenYoav YinonJustin V.C. LemansYochai BirnbaumJustin W. TaylorYogesh BhattaraiJustine FamYohann BohbotJustyna KarolakYohann DabiJutta RichterYohannes W. WoldeamanuelJyoti SharmaYohei MineharuK. Martin KortümYohei SekinoK. RamasamyYoji OguraK. Scott WeberYoko MatsudaK. Thomas RobbinsYonat Shemer-AvniKaboni Whitney GondweYonatan OsterKacper NijakowskiYong AhnKada KloucheYong GeKader ManzurYong Hoon KimKadhim KadhimYong Seuk LeeKai ChenYonggang LiuKai McKeever BullardYongjae YooKai YangYong-Seog OhKaij TreskesYongseok JeeKaining ZhiYong-Soo KwonKai-Wen HsuYong-Sung ChoiKaja MichalczykYong-Yu LiuKajal KhodamoradiYoo Mi KimKajetan GrodeckiYoon Jae LeeKalathil K SureshkumarYoonhyeong ByunKaleen HayesYoon-Ji KimKalla GervasioYoon-Kyoung SungKalyani NairYoon-young ChoiKamal Kant SahuYori GidronKamelia HarrisYorito HattoriKamil JoszkoYoriyasu SuzukiKamil MichalikYoshiaki ShimadaKamil ZaworskiYoshiaki ZaizenKamila KolanskaYoshida HisakoKamila R. IrvineYoshifumi HamasakiKamila SofinskaYoshifumi IkedaKamonwon IenghongYoshifumi KadonoKamran SafaviYoshifumi SaijoKamryn T. EddyYoshifumi SaishoKamyar AsadipooyaYoshiharu KawaguchiKanae TarunoYoshihiko TashiroKang Young ChoiYoshihiko UsuiKankan WangYoshihiro HottaKannamannadiar Jaya-prakasanYoshihiro KinoKanti RaiYoshikane YamauchiKaori NakamuraYoshikazu KobayashiKaori UedaYoshikazu TogoKara GavinYoshiki SeinoKarel TymlYoshimasa AsoKaren BolandYoshimasa HachisuKaren Brandt OnelYoshio YoshidaKaren DoyleYoshiro KodaKaren KafadarYoshiro MatsuiKaren Lindhardt MadsenYoshitaka MatsuuraKaren LindsayYoshiteru MorioKaren SchreiberYoshiyu TakedaKaren SeiterYoshiyuki MishimaKarim DorghamYoshiyuki SueharaKarim HamaouiYosu LuqueKarim HameschYosuke HaradaKarim RehmanYosuke HashimotoKarin WeissenbornYosuke MatsumuraKarina A SerbanYosuke MatsuuraKarina A. Wierzbow-ska-DrabikYoulim KimKarine PanicoYoun Joung ChoKarit ReinsonYoung Goo SongKarl Anders KnutssoYoung Hye ChoKarl Guenter NoéYoung Joo HanKarl Henrik GrinnemoYoung Kyun KimKarl Johan MalmbergYoung Seo ParkKarl NapekoskiYoung Suk KwonKarl SchallerYoung-Jae ChoKarl-Anton HillerYoung-Joon JunKarlijn PellikaanYoung-Min YeKarlmeinrad GiesingerYoung-Ok SonKarl-Michael SchebeschYoung-rak ChoKarol Rawicz-PruszyńskiYoung-Suk LimKarol SzylukYousif SubhiKarolina KossakowskaYoussef HaïkelKarolina KrysinskaYoussef KouidratKarolina M. StepienYoussef OulkhouirKarolina MarkietYo-Wei ChenKarolina MintaYu ChenKarolina SzewczykYu KangKarolina WoronieckaYu KumeKaroline Mayer-PickelYu Kyung ChoKars NevenYu TakahashiKarthik KovvuruYu TaniguchiKarthik RagunathanYuan HuKartik AnandYu-Ching ChouKasper G LauridsenYue ShengKassiani TheodorakiYuhi FujimotoKatarina DakayYuh-Mou SueKatarina Steding-EhrenborgYuh-Shyan TsaiKatarina StinglYuichi InoueKatarzyna GórskaYuichi SaitoKatarzyna GrocholewiczYuichiro IkebuchiKatarzyna HojanYuichiro NishidaKatarzyna HolcmanYuji NadataniKatarzyna Kiliś-PstrusińskaYuji NagatomoKatarzyna KotfisYujiro SanoKatarzyna KoziakYu-Jung ChengKatarzyna KrysikYukari Aoki‐NonakaKatarzyna LomperYuki KataokaKatarzyna Michalska-MałeckaYuki SaitoKatarzyna Mocny-PachońskaYuki YamashitaKatarzyna Mozolewska-PiotrowskaYukiko KanoKatarzyna NowomiejskaYukiko OkamiKatarzyna PawlakYukinari KakizawaKatarzyna PiwockaYukio MaruyamaKatarzyna Ptaszyn-ska-KopczynskaYukio SekiKatarzyna StarowiczYukiyo ShimizuKatarzyna WalickaYuko OnoKatarzyna Wróblew-ska-SeniukYuma FuseKate MaslinYumi MochizukiKatelyn FrenisYun HanKaterina GrafanakiYun Nah LeeKaterina KambouriYungi KimKaterina LaskariYung-Tsu ChoKaterina TheodoropoulouYunhua Li MullerKaterina VaporidiYunis M. MayasiKatharina BeckYun-Wen ZhengKatharina KählerYURI BattagliaKatharina KurzYusuke KatayamaKatharina SommerYuta TerabeKatharine M. LodgeYutaka TojoKatherine A. GiulianoYutaka UedaKatherine BiancoYuto UedaKatherine E. GoodmanYuzuru SakamotoKatherine E. McDonellYves BorbélyKatherine H. IngramYves GouriouKatherine KnoxYves HélouryKatherine M. HammittYvonne BrysonKatherine Murray-LeisureYvonne Jockel-SchneiderKatherine OgurtsovaYvonne NitschkeKathleen A. R. SchildrothZ. BergerKathleen HaleyZaher BahouthKathleen NichollsZahra AshenaKathleen PuntilloZaid Al-QurayshiKathrin JobskiZaneta Kimber-TrojnarKathrin MalejkoZawiejska AgnieszkaKathrin NickelZaza KatsaravaKathrin SeveckeZbigniew AdamiakKathryn BowlesZbigniew KrasińskiKathryn L. Van PeltZbyněk StraňákKathryn NicholsonZeev OrmianerKathryn Y. BurgeZehava S. RosenbergKathy BondZelazny JamieKatia BoggianŽeljka Večerić-HalerKatia OrvinZenithson Y. NgKatie L. PenningtonZenon MariakKatie PageZenon PogorelićKatie SmithZeribe Chike NwosuKatie WynneZhanqi ZhaoKatja SchladitzZhe LiKatja Van Den HurkZhejun WangKatrin Anne BeckerZhen ZhouKatrin D. Bartl-PokornyZheng Zachory WeiKatrin OttersbachZhengwang SunKatrin Streckfuss-BömekeZhi ZhangKatsuhiro ItoZhi-Chun DingKatsuki TsuchiyamaZhihong YangKatsunori IijimaZhijuan QiuKatsunori UchidaZhipeng LiuKatsushi TakebayashiZhiqiang KuKatsuyuki MiyabeZhirong YangKaty PikeZhongqiu XieKaustubh R. PatilZiethe AnkeKaustuv BhattacharyaZin Z. KhaingKaveh EghbalzadehZingaro Michele DelKaweh MansouriZiolkowska LidiaKayleigh MasonZoe HallKaylena Ehgoetz MartensZoe RutherfordKayo TanakaZofia T. BilińskaKazimierz KuliczkowskiZograb MakiyanKazuaki YoshimuneZohaib YousafKazuhide OhtaZoilo MadrazoKazuhiko ArimaZoltán CsanádiKazuhiro MoriokaZoltan SzekaneczKazuho HondaZsófia BenkőKazuki M. MatsudaZsombor LaczaKazunari FushimiZsuzsa SchaffKazunari NakaharaZsuzsanna RecsanKazunori KawaguchiZubair BalochKazuo KitamuraZuleika Saz-ParkinsonKazuo TakahashiZul’Fiya Sagdullaevna Kho-dzhaevaKazushige NireiZuyue WangKazushige OshitaZyanya Reyes-Castillo


